# Supplementary material for: Automated task training and longitudinal monitoring of mouse mesoscale cortical circuits using home cages
Source: eLife. 2020 May 15;9:e55964. doi: 10.7554/eLife.55964 (PMC7332290; doi:10.7554/eLife.55964)
Supplement: Supplementary file 1. [file elife-55964-supp1.pdf]

Please cite Murphy et al. 2020 eLife

Table of Contents

Cages 3
Part List 3
Hardware Parts 3
Machined Parts (Stainless Steel) 3
3D Printed (Black PLA) 3
3D Printed (Protolabs Watershed) 4
Electronics 4
Assembly 5
Cage Cut-outs 5
Head-fixing Chamber Setup 7
Load Cell Attachment 12
RFID Tag Reader Setup 13
Brain Imaging Camera 15
IR Beam & Piezo Speaker and Vibrating Motor 16
Water Reward System 16
Behaviour Cameras 19
Attaching Triple LED System 22
Triple LED Light Guide 23
Part List 23
Hardware Parts 23
Machined Parts (Stainless Steel) 23
3D Printed (Black PLA) 23
Optics 23
LEDs 24
Electronics 24
Assembly 25
LEDs 25
LED Housings 26
Dichroic Mirror Housing 26
Electronics 28
Main Cage Wiring 28

Connections 28
Electronic Box and Extension Breakout 28
Auto-Weighing Tunnel 29
Behaviour Cameras 29
Triggered Recordings 29

Cages

Part List

Hardware Parts

Description	Manufacturer	Part Number	Quantity
1/4-20 Bolts, Setscrews, Nuts, Washers			
8/32 Bolts, Nuts, Washers			
M2 & M2.5 Bolts, Nuts, Washers			
Cage 7.5" X 11.5" X 5"	Lab Products	10027	2
Aluminum Breadboard 18" x 24" x 1/2", 1/4"-20 Taps	Thorlabs	MB1824	1
Ø1" Pillar Posts with 1/4"-20 Taps, 6"	Thorlabs	RS6	10
Ø1" Pillar Posts with 1/4"-20 Taps, 3"	Thorlabs	RS3	8
Ø1" Pillar Posts with 1/4"-20 Taps, 2"	Thorlabs	RS2	4
Clamping Fork, 1.24" Counterbored Slot, Universal	Thorlabs	CF125	4
Ø1/2" Pedestal Post Holder, Spring-Loaded Hex-Locking Thumbscrew, L=2.19"	Thorlabs	PH2E	4
Ø1/2" Optical Post, SS, 8-32 Setscrew, 1/4"-20 Tap, L = 8"	Thorlabs	TR8	3
Ø1/2" Optical Post, SS, 8-32 Setscrew, 1/4"-20 Tap, L = 12"	Thorlabs	TR12	2
Right-Angle Clamp for Ø1/2" Posts, 3/16" Hex	Thorlabs	RA90	4
Ø25 mm Post Spacer, Thickness = 3 mm	Thorlabs	RS3M	1
Glass cut to 93mm X 31mm X 3mm rectangle	N/A	N/A	1

Machined Parts (Stainless Steel)

File Name	Quantity
Head_fix_servo_coupler_PROFESH_revised_no_pinch.ipt	1
ahf_contact_plate_L.stp	1
ahf_contact_plate_R.stp	1

3D Printed (Black PLA)

File Name	Quantity
AHF_camera_mount_1p_EXTENDED.stl	1
Camera_Mount_V2.stl	3
Tunnel_Coupler.stl	1
Tunnel_Guider.stl	2
Tunnel_V2.stl	1
RFID_Holder.stl	1

3D Printed (Protolabs Watershed)

File Name	Quantity
head_bar_grabbing_plate_extended_6mm_hole_in_bottom V24 barrier1mmgreater_cut_front_bottom.stl	1

Electronics

Description	Manufacturer	Part Number	Quantity
Stranded wire, Solder, Flux	N/A	N/A	
SD-80LP Plug	Digikey	CP-1038-ND	1
JST Connector 2 & 3 positions, 2.5mm pitch	Digikey	455-2247-ND<br>455-2248-ND	2p: 3<br>3p: 1
PTPM Connector 2 positions, 2.5mm pitch	Phoenix Contact	1780477	2
HS-645MG High Torque, Metal Gear Premium Sport Servo	Hitec	32645S	1
RFID Reader ID-20LA (125 kHz)	ID-Innovations	ID-20LA	2
RFID Reader Breakout	Sparkfun	13030	2
Solenoid Valve	Gems Sensors	MB202-VB30-L203	1
IR Break Beam Sensor - 5mm LEDs	Adafruit	2168	1
RPi Camera (F), Supports Night Vision, Adjustable-Focus	Waveshare	10299	4
Flex Cable for Raspberry Pi Camera or Display - 2 meters	Adafruit	2144	4
Load Cell Amplifier - HX711	Sparkfun	SEN-13879	1
Micro Load Cell (0-100g) - CZL639HD	Bonad/Alibaba	CZL639M	1
Vibrating Mini Motor D	Adafruit	1201	1
Small Wire Piezo	Adafruit	1740	1
Lever Pulling Task Device	N/A	N/A	1
Lever Pulling Task Device Extension	N/A	N/A	1

Assembly

Cage Cut-outs

The first two cut-outs will be on the sides of the two cages so a tunnel can be attached between them. This will be used for automatic weighing of the mice (manual weighing and supplementing was still conducted). Mirror the cut-out on the cage below for the right cage.

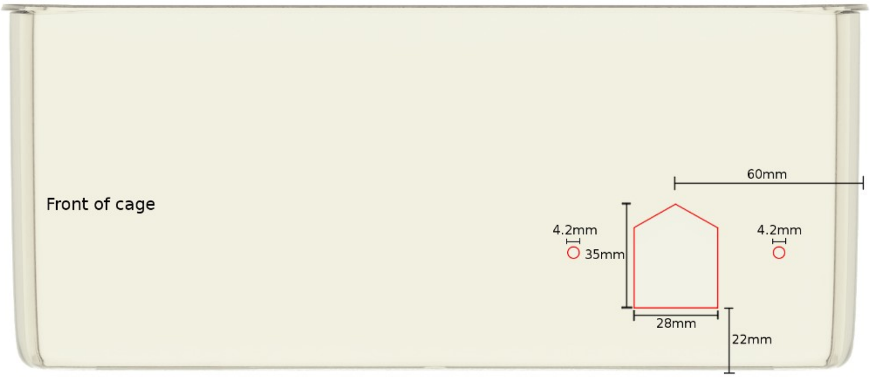

Placement of the circular holes for the bolts attaching the Tunnel Guideway piece can be found by overlaying the printed piece and marking the place where they fall (approx 35mm from center).

Notice the placement of the guide: Ensure enough space is left on all four sides for the guideway to float.

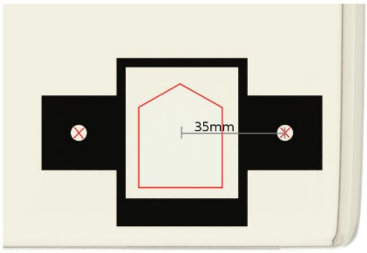

The Second cut-out will be on the front side of the left cage and will be used to attach the auto head-fixing chamber and camera.

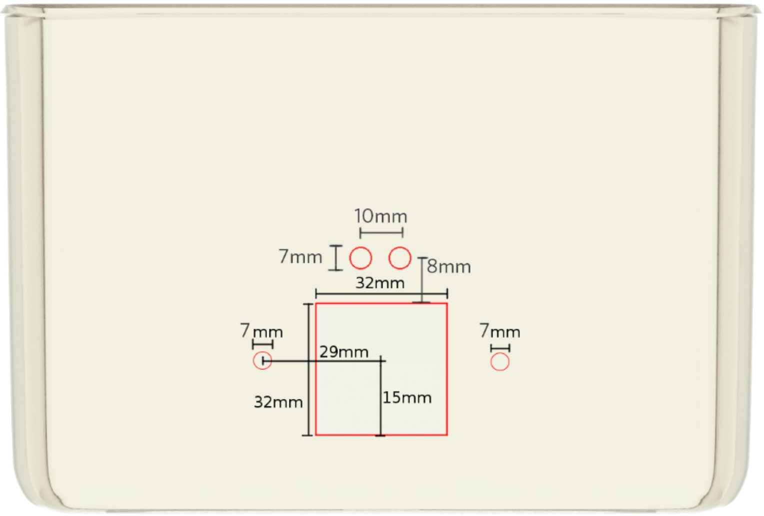

Next drill the holes to attach posts to the cages using ¼-20 bolts as shown below.

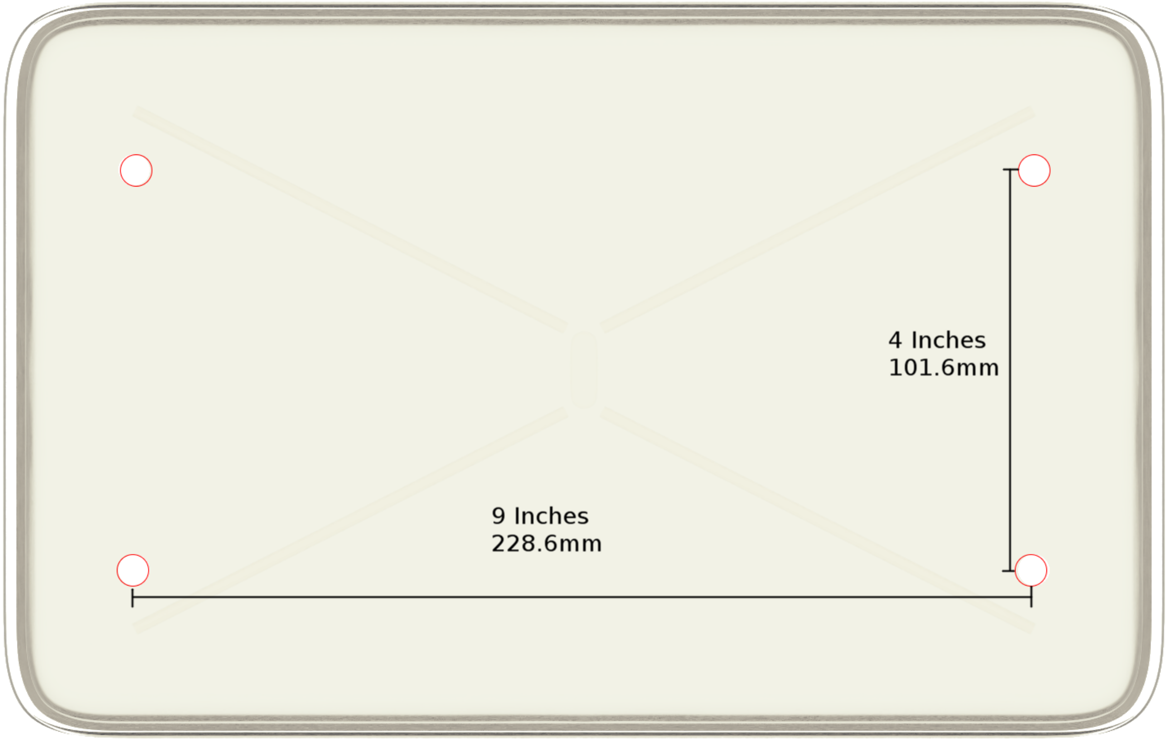

The figure below shows all cut-outs.

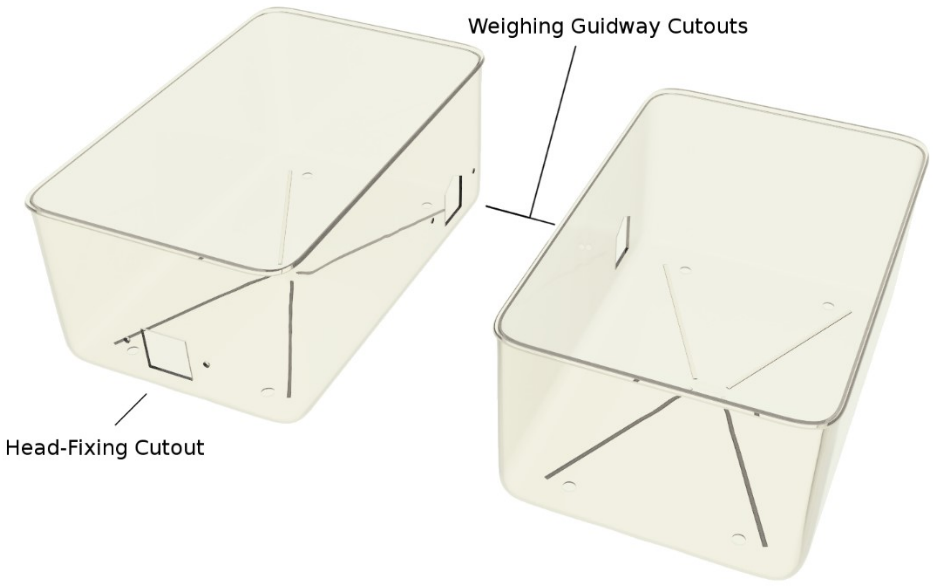

Head-fixing Chamber Setup

-The chamber consists of:

- head_bar_grabbing_plate_extended_6mm_hole_in_bottom V24 barrier1mmgreater_cut_front_bottom.stl
- Head_fix_servo_coupler_PROFESH_revised_no_pinch.ipt
- ahf_contact_plate_L.stp
- ahf_contact_plate_R.stp
- Glass cut to 93mm X 31mm X 3mm rectangle

First add the rubber spacers to the motor (labeled 1), then cut the motor arm (labeled 2) to only include the top two holes as illustrated below. Make sure to shave the sides as much as possible so head-fix coupler can rotate as freely as possible. Finally, attach the head-fix coupler to the lowest hole and secure it using a brass rod cut to size (rod diameter ~1.5mm). Secure T-piece with 1.5 mm bronze rod (or a syringe needle) that passes through the T-piece and bisects the servo arm (#2). It is critical to trim the bronze rod so there is no protruding pieces that can catch the headfixing tube, add Krazy glue to secure the rod.

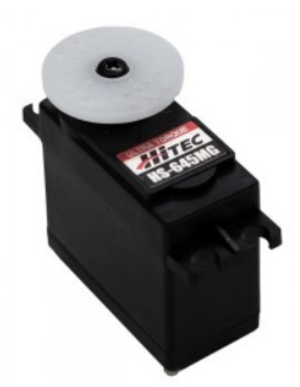

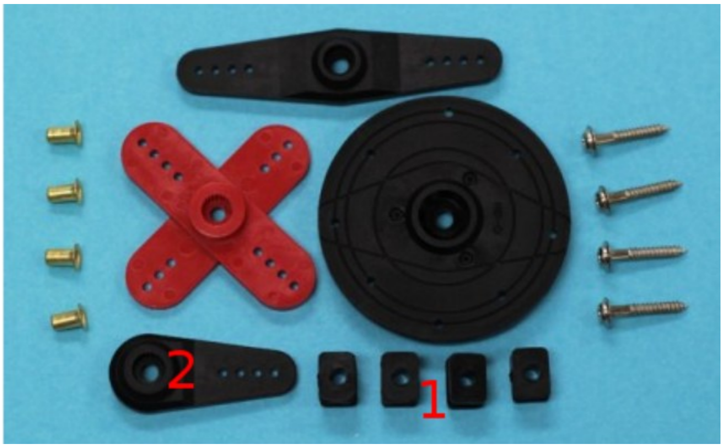

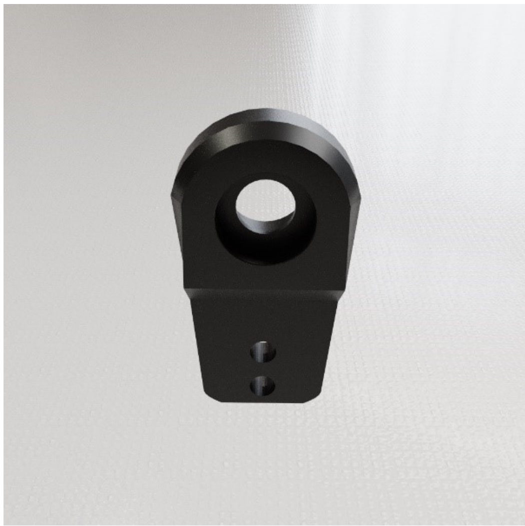

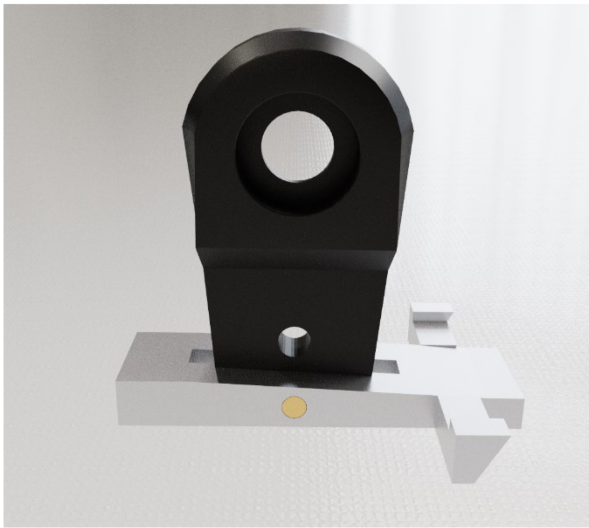

Attach the servo motor onto the printed chamber using 4 M2.5 bolts and nuts. Then slip the motor arm with the coupler through the bottom and attach it to the servo. Then bolt onto the chamber the two contact plates using M2.5 bolts and nuts with washers. Finally glue the glass piece using holt melt glue to create a floor. Bronze securing rod seen on right image above.

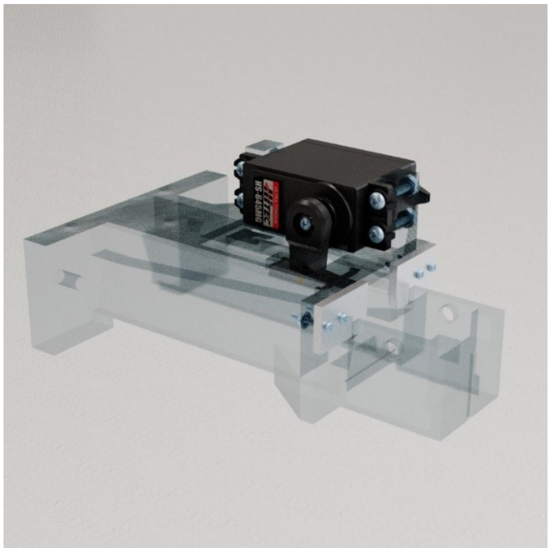

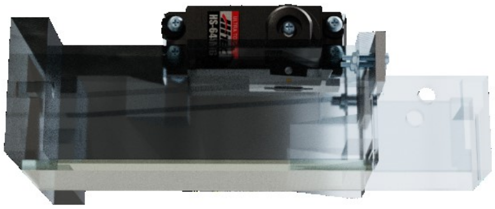

Drill a 1mm hole at the front of the chamber. This will be used to slip the water spout/needle in.

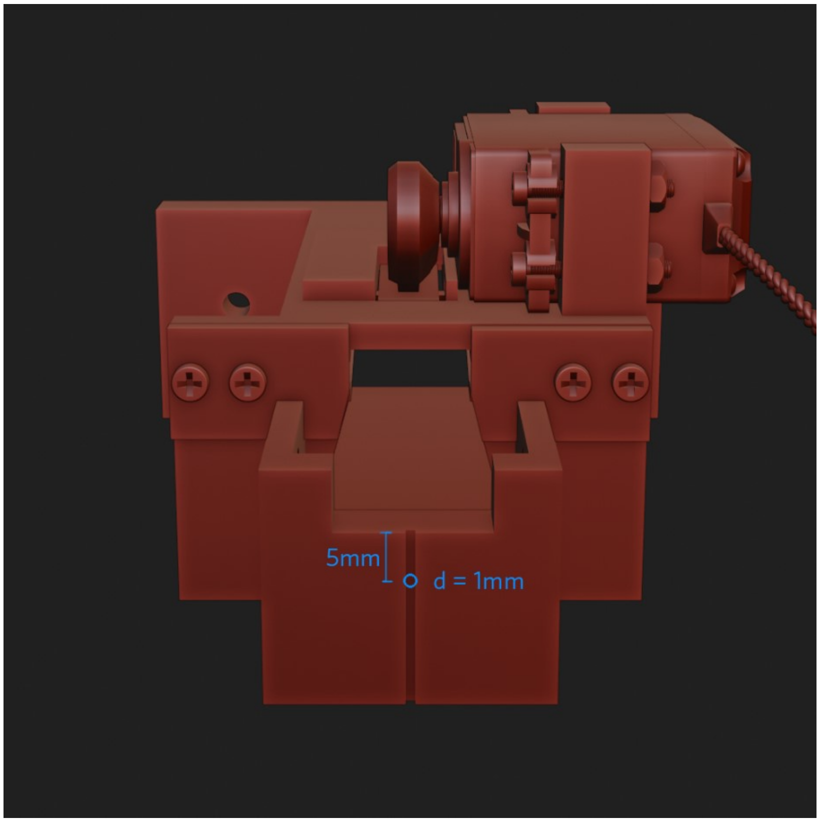

Attach the chamber onto the cage using ½-20 bolts, nuts, and washers. Then add the tunnel guideway pieces onto the two side cut outs using 8-32 bolts, nuts, and washers.

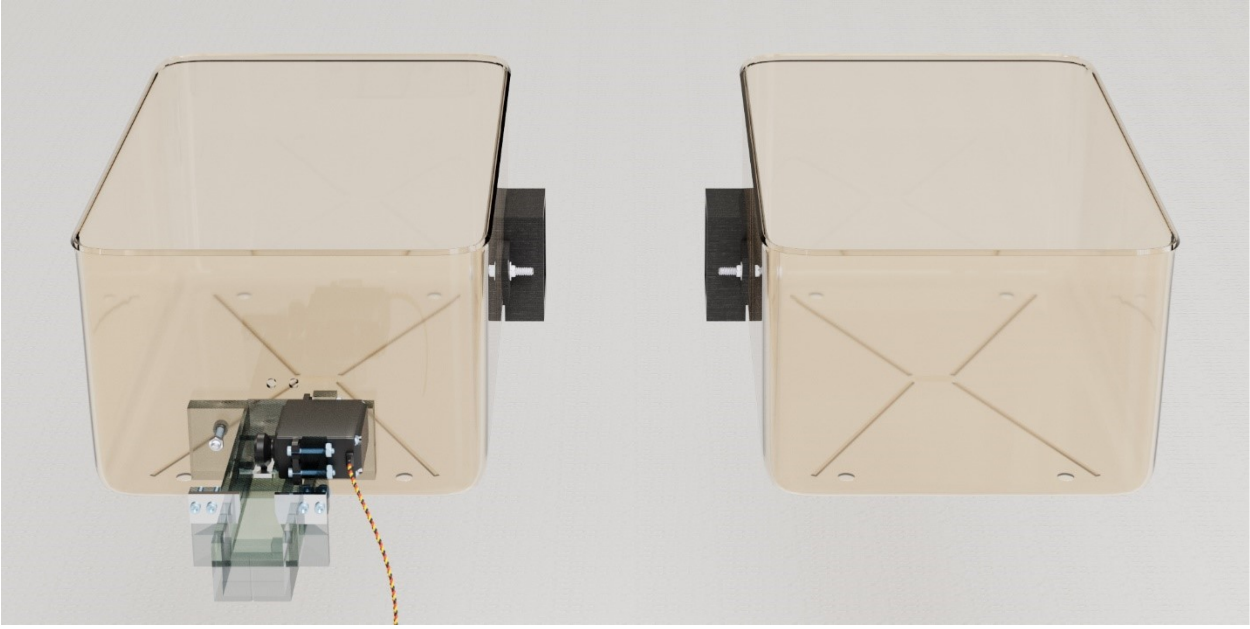

Breadboard Setup

The aluminium breadboard will hold all components to make transportation and secure attachments easier. First add four 2.0” pillars to the four corners in the counter-bored holes, this will make picking up the breadboard easier.

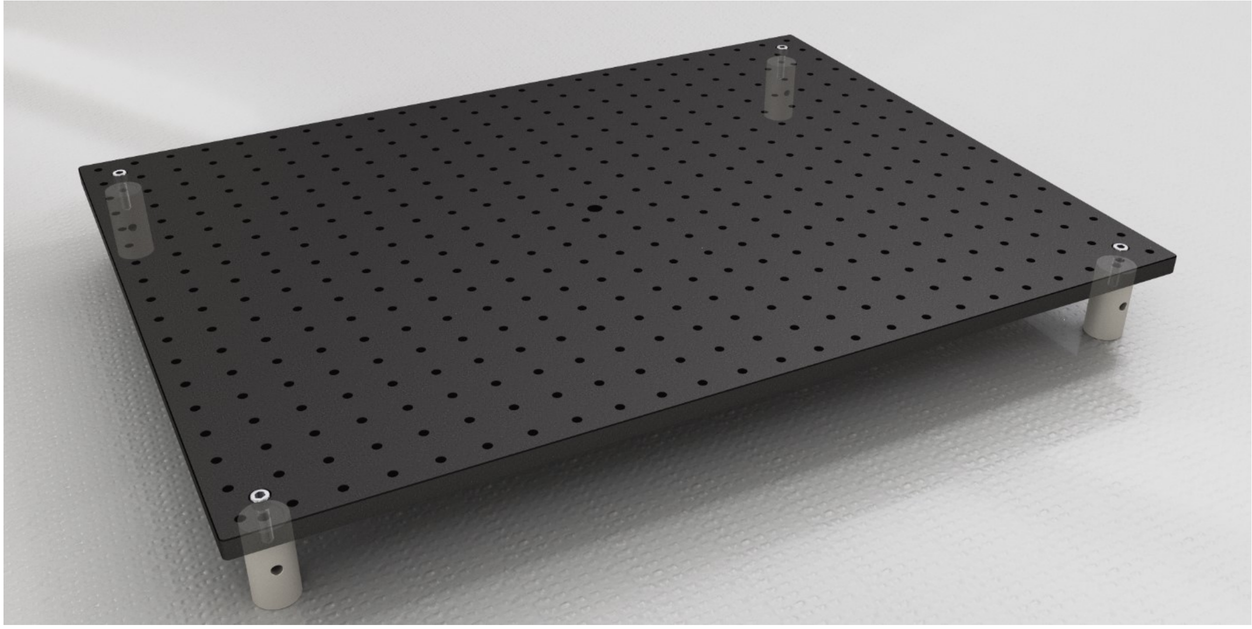

Next, connect 8 pairs of the six and three inch pillars using setscrews. These will be used to elevate the cages. Attach them to the breadboard using setscrews as shown below.

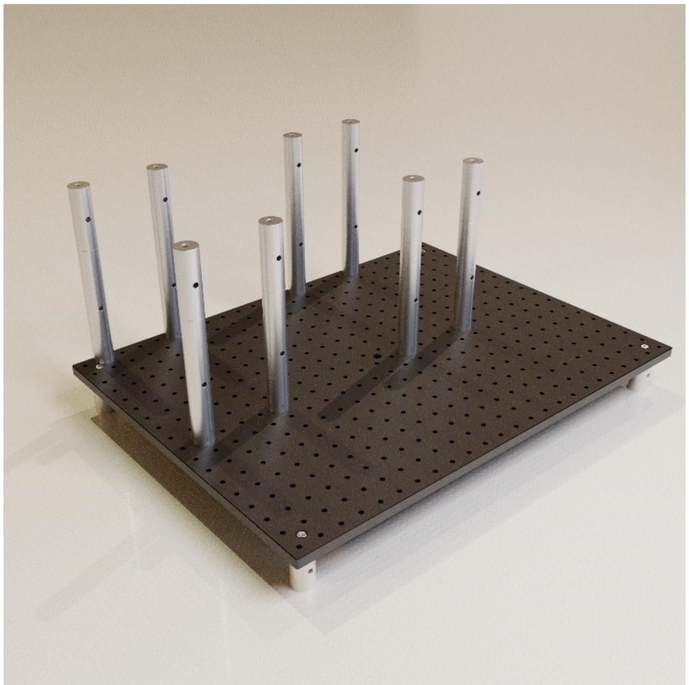

Bolt onto the pillars the two cages with the tunnel guideways and chamber attached, ensuring the two weighing tunnel cut-outs are facing each other. After bolting one cage, slip the weighing tunnel into one of the guides and bolt the other cage.

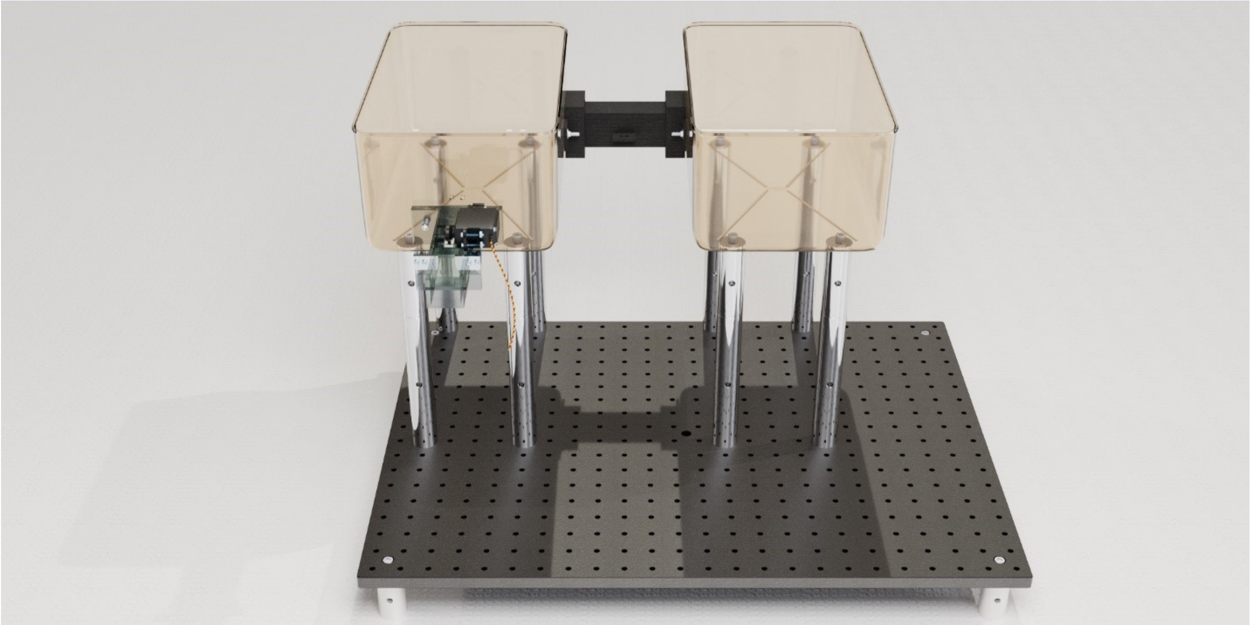

Load Cell Attachment

The load cell requires that their wires be extended. Add heat-shrinking tube to the wires and solder onto them longer and thicker wires ~0.5m long. Be careful as the wires are extremely delicate and can be ripped off easily. Attach the load cell onto the tunnel coupler using M2 screws and nuts. Then using the 1/8” pillar as a spacer attach an 8” post using an 8/32 screw. Then add a post holder and a clamping fork and attach to the breadboard securing it to the tunnel itself using M2 screws and nuts.

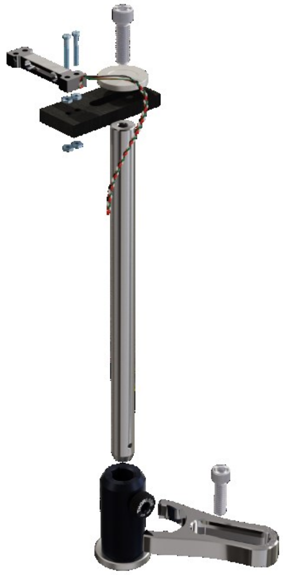

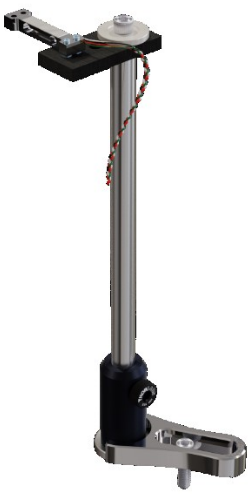

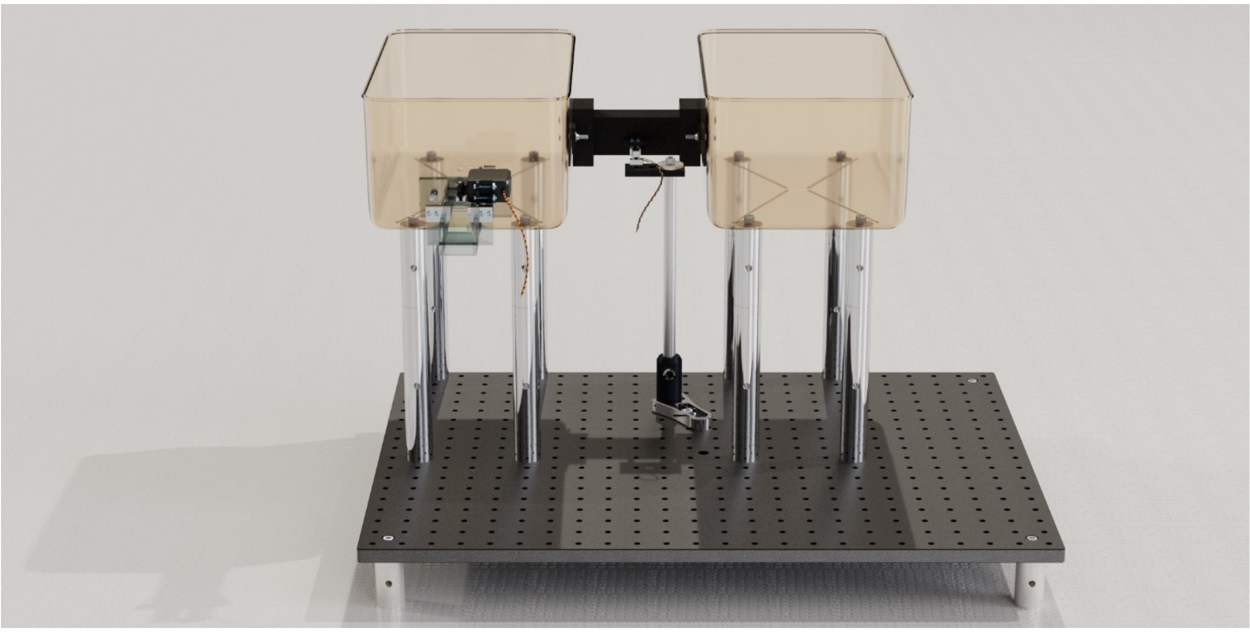

RFID Tag Reader Setup

Two RFID tag readers will be made. One will be used for the weighing tunnel and the other for the head-fixing chamber. To get them ready, solder the RFID breakout board onto the ID20-LA antenna. Then two interconnections will be made using stranded wire (VCC-RES & GND-FORM) and four wires will be made into a cable that connect to VCC, GND, D0 & TIR.

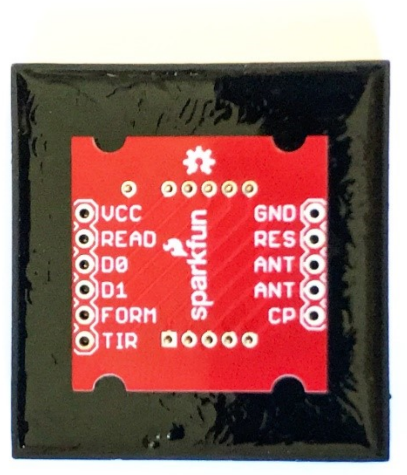

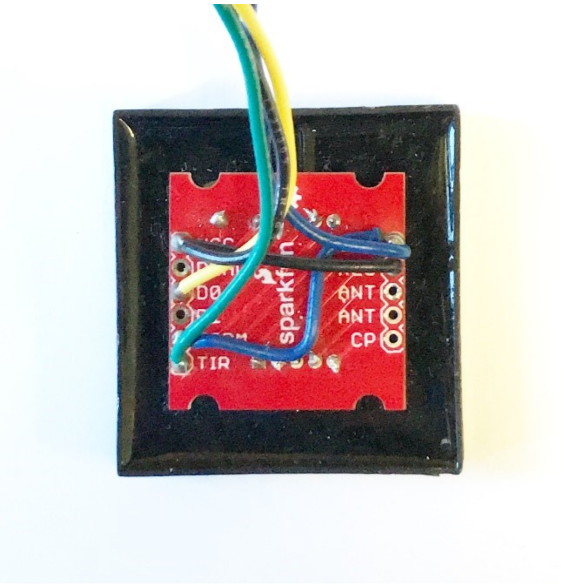

One of the RFID boards will plug into the Lever Pulling Task box. The connection is made using the SD-80LP plug. Solder the wires to their corresponding pins as shown below. The other will be plugged in directly to a Raspberry Pi board for the automatic weighing.

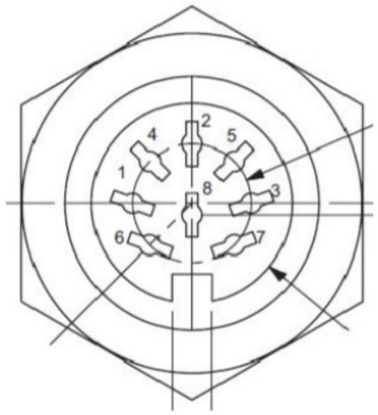

Pin 1: D0-Data
Pin 2: TIR Tag in Range
Pin 3: Read
Pin 4: +5V VCC
Pin 5: GND
Pin 6: Antenna1 (Internal)
Pin 7: Antenna2 (Internal)
Pin 8: Shield

The RFID with the SD-80LP plug will be glued onto the chamber's glass floor using hot melt glue. The weighing tunnel RFID will require a holder. Using the 3D printed RFID holder attach a post using an 8-32 screw. Then attach the post to the post holder and clamp it using a fork.

Assembly of the RFID holder.

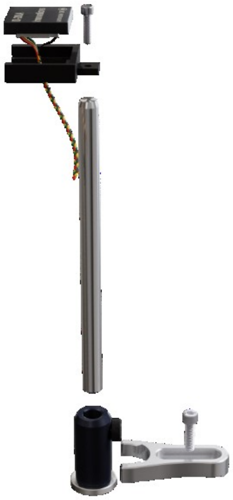

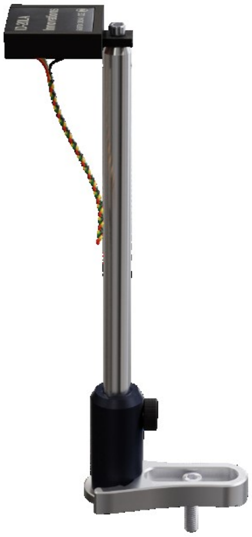

RFID with holder is slipped underneath the weighing tunnel.

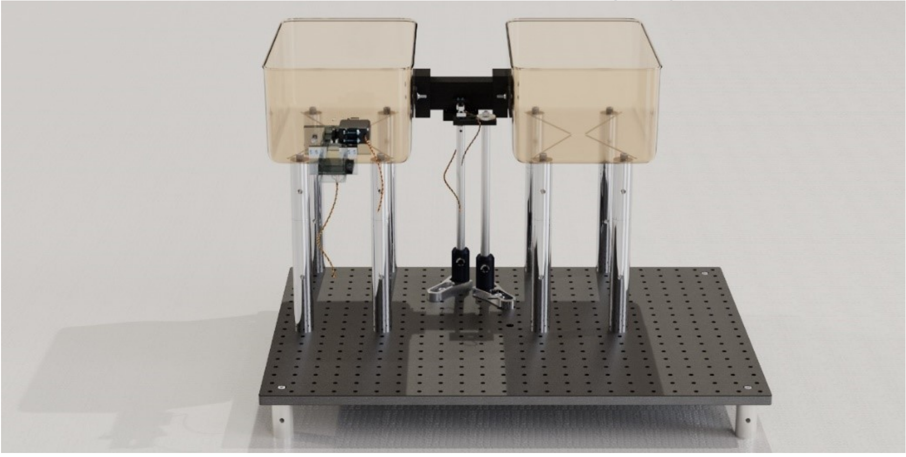

The second RFID is glued onto the floor of the chamber using hot melt glue.

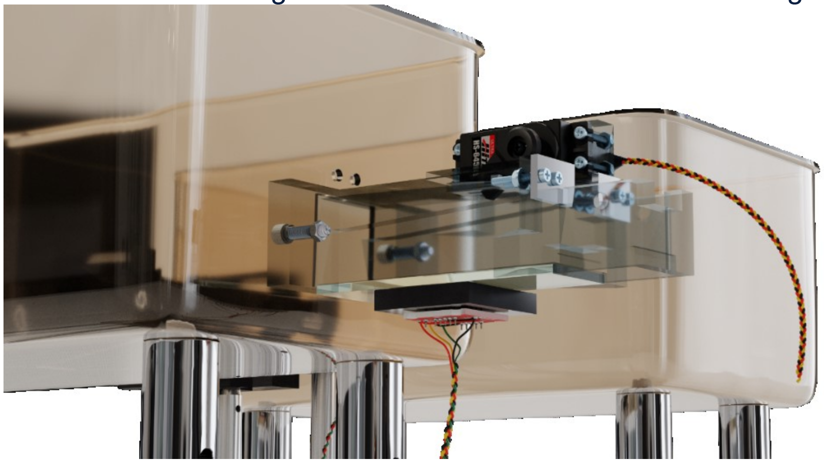

Brain Imaging Camera

To get the camera ready, we must add the IR filter onto the sensor using superglue. Carefully cut the filter to fit over the sensor. Then super glue the triple band-pass filter onto the bottom of the lens using a single drop of super glue. If the lens/filter get a small amount of superglue, separate the pieces and use acetone to clean them and try again.

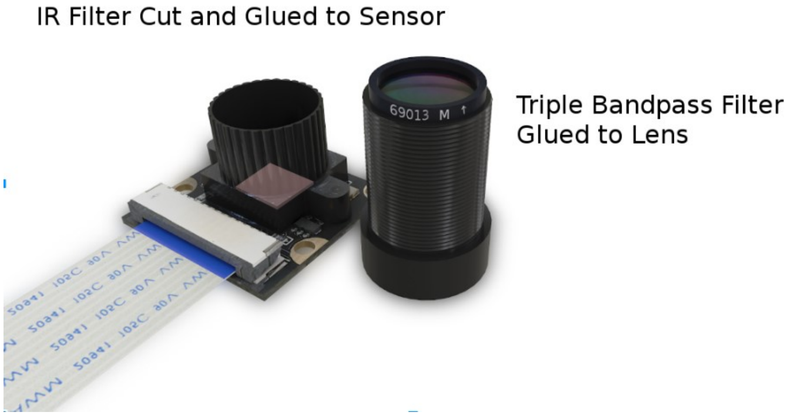

Next, screw the camera board onto the camera holder printed piece using M2 screws and nuts, and change the provided ribbon cable to the long one. Slip the ribbon cable through the slit and attach the camera holder onto the cage using ¼-20 bolts, nuts, and washers.

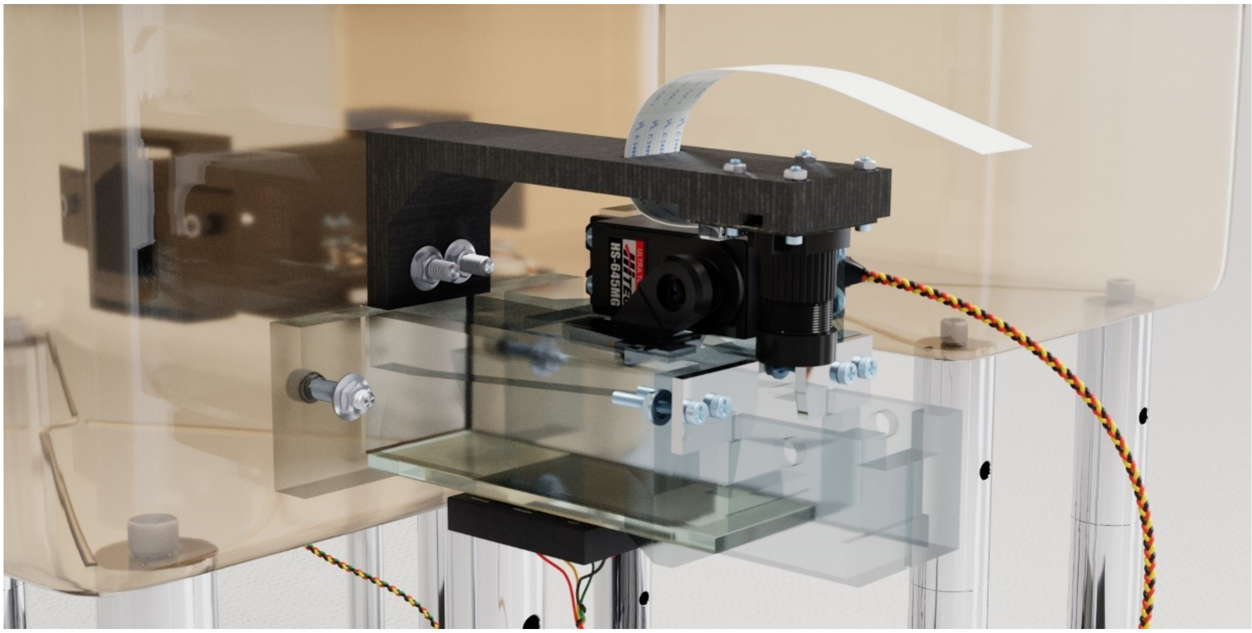

IR Beam & Piezo Speaker and Vibrating Motor

Extend the wires for each piece using heat-shrinking tube as well. The IR beam sensor slips onto the holes at the front of the chamber and can be secured using hot melt glue. The piezo speaker and motor can be placed below the floor as far forward as they can go and be glued on as well.

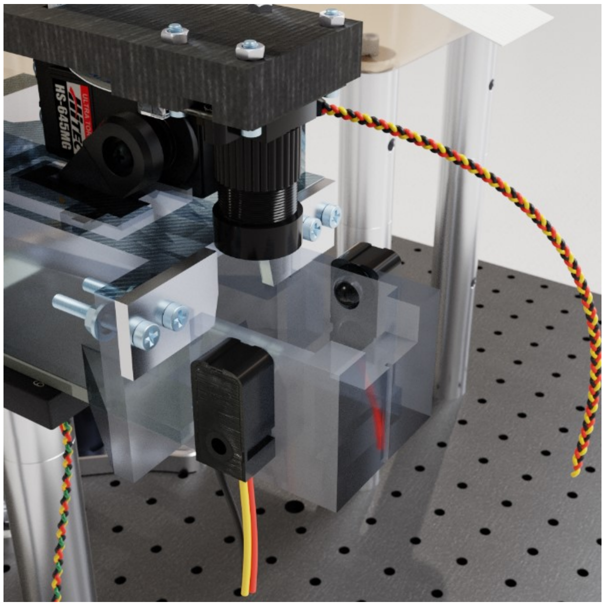

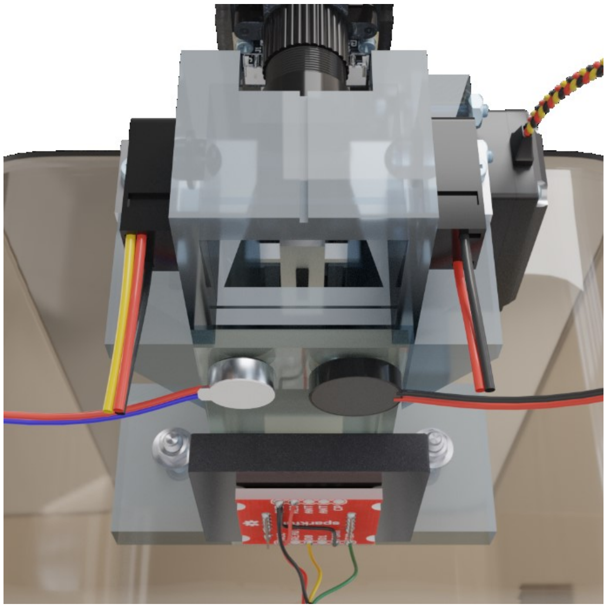

Water Reward System

Water is dispensed using a solenoid. As usual, extend the solenoid's wires then attach two 6" pillars onto the breadboard with setscrews. Attach the bottle holder using a ¼-20 bolt as shown below.

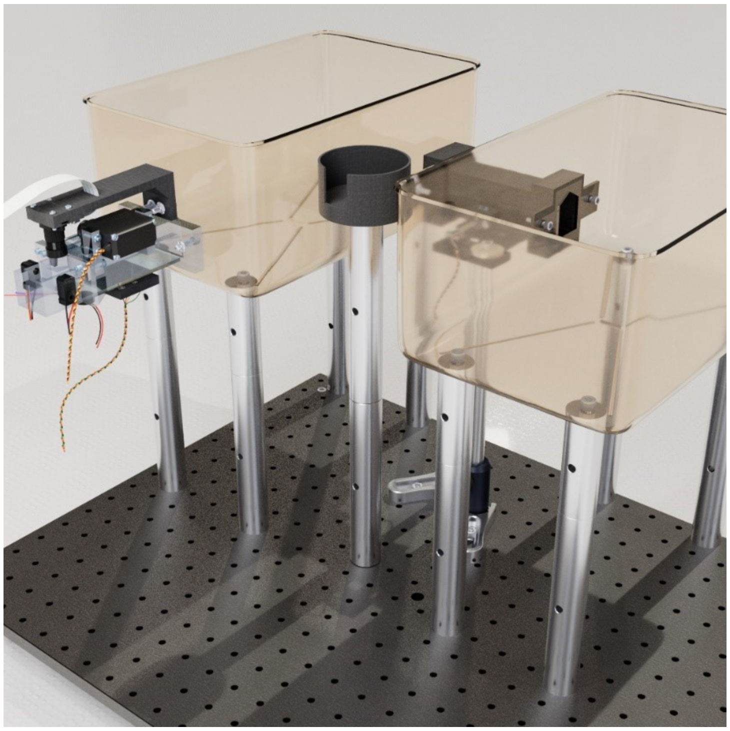

Drill a hole to fit the small end of a male luer inside the water bottle with the big end on the inside to create a seal, gluing it in place using hot melt glue. Attach piping and attach it to the valve closest to the casing of the solenoid. Another pipe is attached to the other valve and using another luer convert to smaller piping which attaches to the 22-gauge needle. The needle is cut to ¾ of an inch in length and filed down to not hurt the mouse if it gets poked. Be careful as cutting the needle can cause it to cave in and create a seal. If that happens take some pliers and open the tip up again. The needle is then put through the 1mm hole drilled at the chamber's front wall. The bottle can then rest on the bottle holder and the solenoid can be attached to the pillars using Velcro or tape.

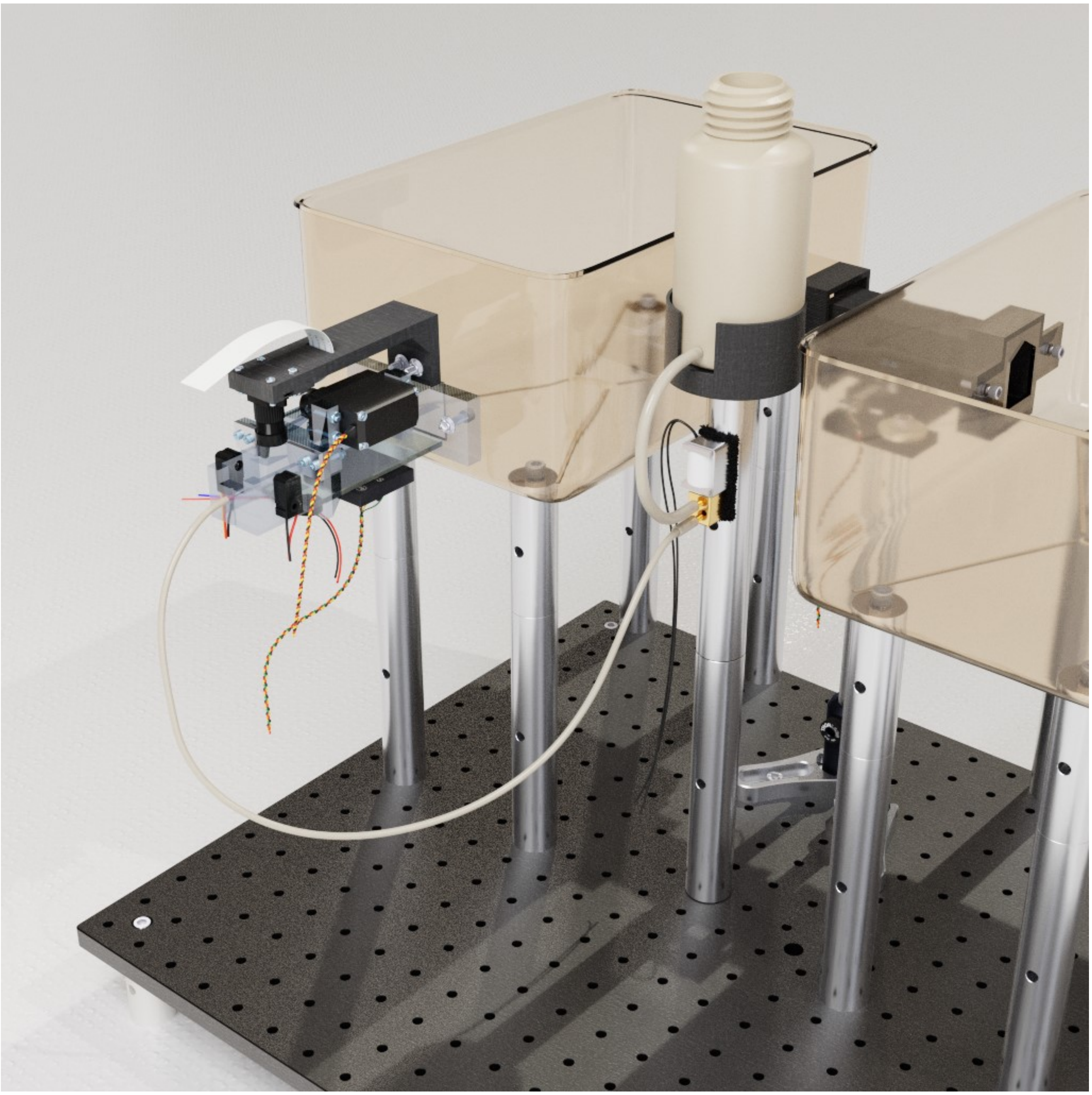

Behaviour Cameras

We use three behaviour cameras to record regions of interest. A forward-facing camera mounted beneath the triple LED light guide will be used to record whisker and snout movement. A camera mounted on the side is used to record eye behaviour. Finally, a third camera is mounted beneath the chamber to record hind and forelimb movement.

The forward-facing camera and the side camera are built using the camera_mount_v2 printed piece and 90-degree angle clamps attached to posts that connect to a post holder that is fixed using a fork clamp as shown below.

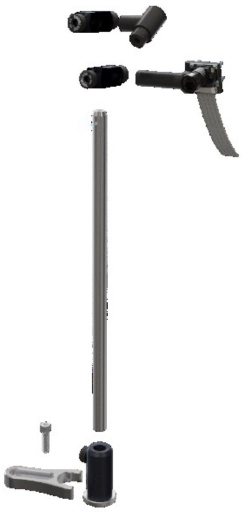

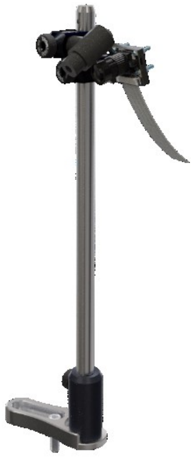

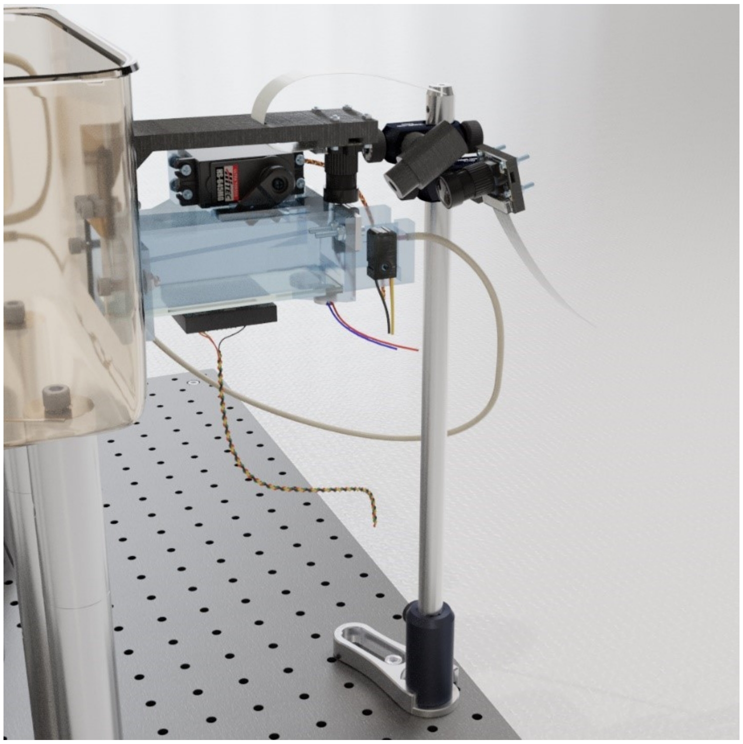

Eye Camera and Body Camera Assembly:

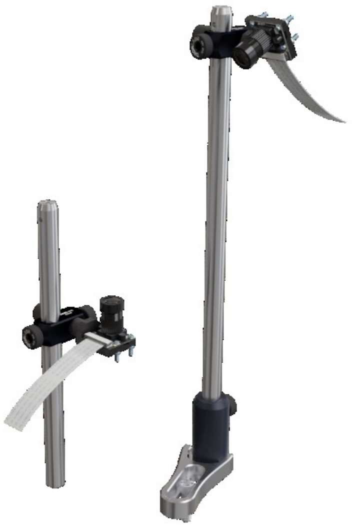

Body camera placed to look up at the chamber.

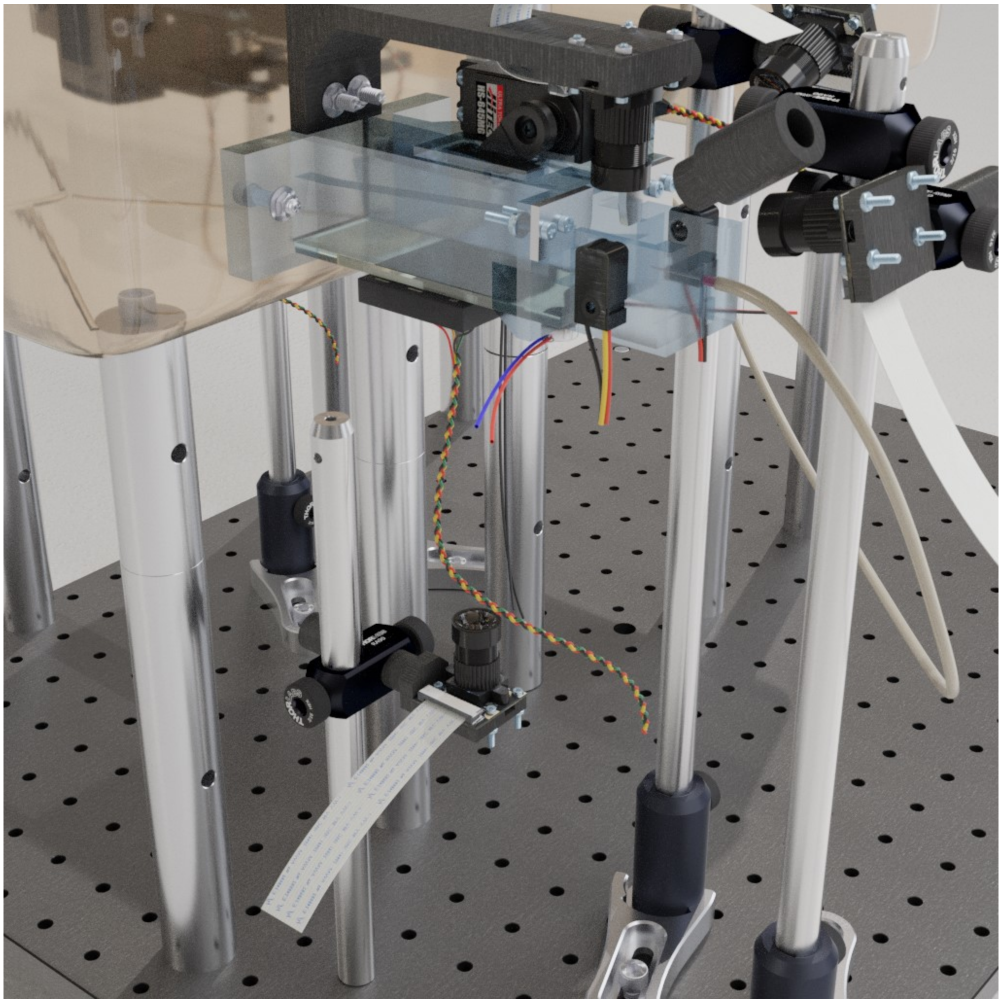
Eye camera placed adjacent to liquid light guide holder and front-facing camera.

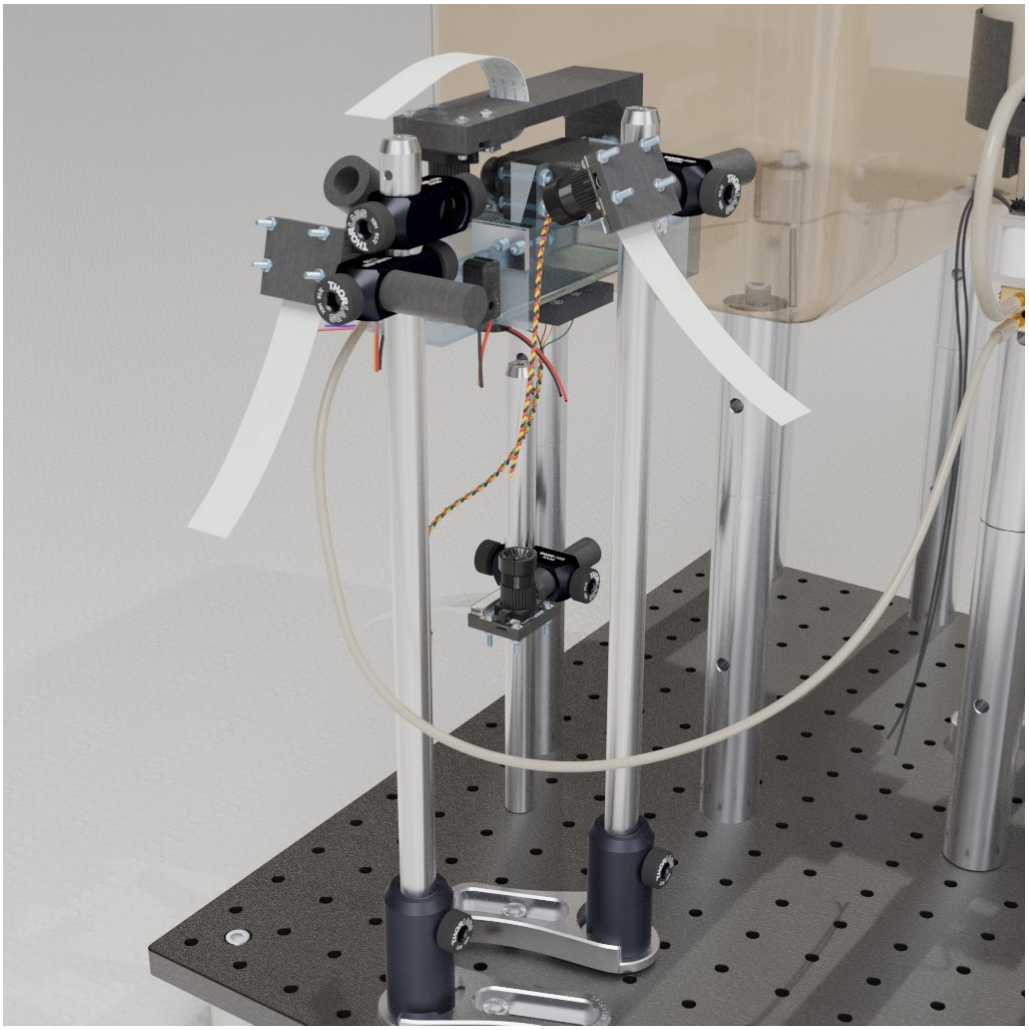

Attaching Triple LED System

Finally, build a triple LED system as described below, and attach the liquid light guide to the holder. The holder may need to be filed and expanded using a drill to have a secure placement without it being able to move freely.

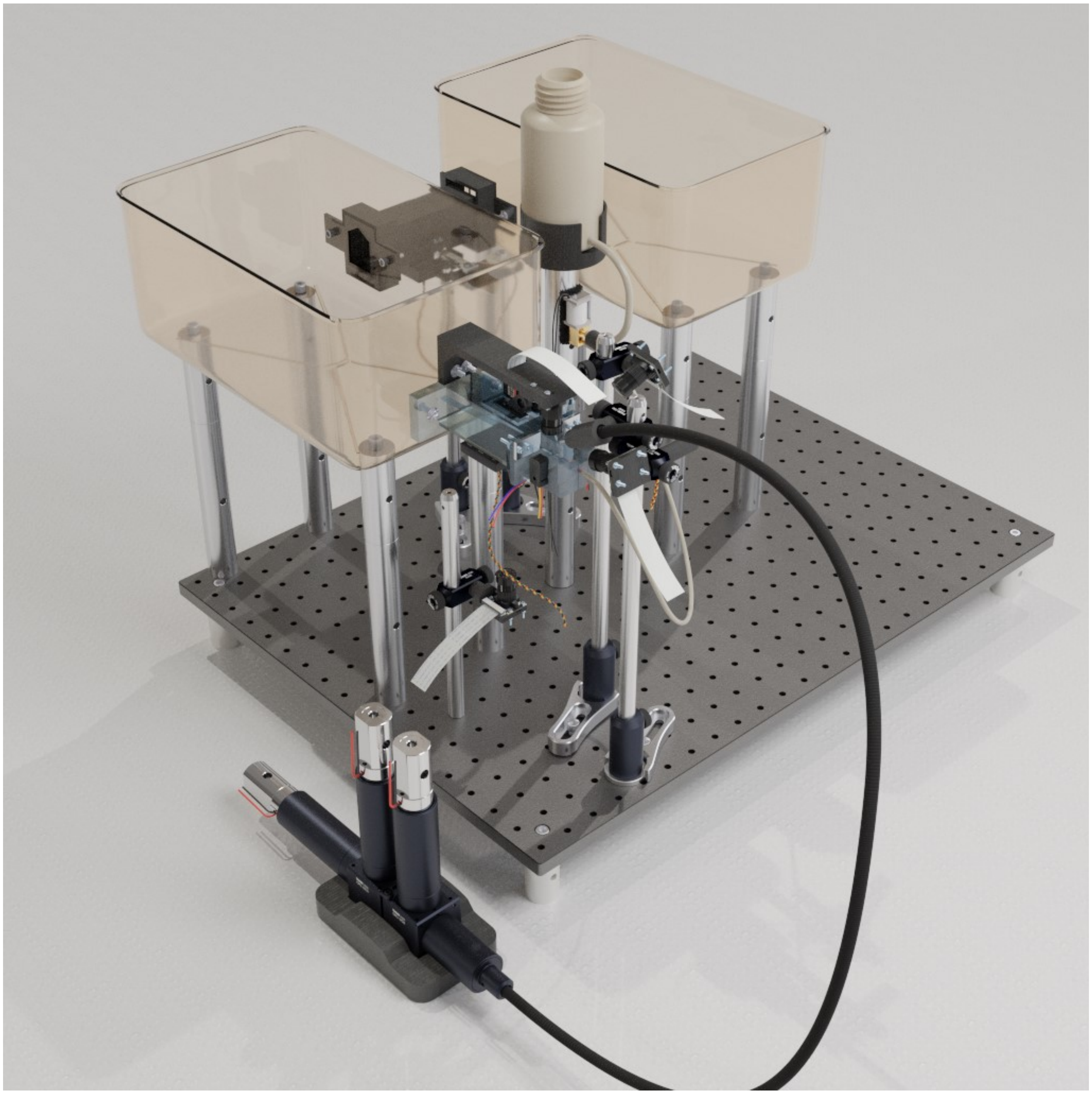

Triple LED Light Guide

Part List

Hardware Parts

Description	Manufacturer	Part Number	Quantity
Liquid Light Guide Ø3 mm Core, 4' (1.2 m) Length	Thorlabs	LLG0338-4	1
Ø3 mm LLG to SM1 Adapter	Thorlabs	AD3LLG	1
SM1 Lens Tube, 3.00" Thread Depth, One Retaining Ring Included	Thorlabs	SM1L30	3
SM1 Lens Tube, 0.50" Thread Depth, One Retaining Ring Included	Thorlabs	SM1L05	3
SM1 Lens Tube, 2.00" Thread Depth, One Retaining Ring Included	Thorlabs	SM1L30	1
30 mm Cage Cube with Dichroic Filter Mount	Thorlabs	CM1-DCH	2
Cage Cube Connector for Compact 30 mm Cage Cubes	Thorlabs	CM1-CC	1

Machined Parts (Stainless Steel)

File Name	Quantity
Milled as-1.50_2_v2.SLDPRT	3
Spacer_with_wire_hole_as-.500_v2.SLDPRT	3
LED_mount_as-1.50_v2.SLDPRT	3

3D Printed (Black PLA)

File Name	Quantity
TripleLEDLightGuide_Base.stl	1

Optics

Description	Manufacturer	Part Number	Quantity
AT455DC Size: 26 * 38 mm	Chroma	AT455DC	1
25 mm x 36 mm Longpass Dichroic Mirror, 550 nm Cutoff	Thorlabs	DMLP550R	1
Ø1" Bandpass Filter, CWL = 630 ± 2 nm, FWHM = 10 ± 2 nm	Thorlabs	FB630-10	1
Ø1" Bandpass Filter, CWL = 440 ± 2 nm, FWHM = 10 ± 2 nm	Thorlabs	FB440-10	1
ET480/30x Size: 25mmR R=Mounted in Ring	Chroma	ET380/30X	1
N-BK7 Bi-Convex Lens, Ø1", f = 25.4 mm, Uncoated	Thorlabs	LB1761	4

LEDs

Description	Manufacturer	Part Number	Quantity
Royal-Blue (448nm) Rebel LED on a SinkPAD-II 20mm Star Base - 1030 mW @ 700mA	Luxeonstar	SP-01-V4	1
Blue (470nm) Rebel LED on a SinkPAD-II 10mm Square Base - 65 lm @ 700mA	Luxeonstar	SP-05-B4	1
Red (627nm) Rebel LED on a SinkPAD-II 20mm Star Base - 64 lm @ 350mA	Luxeonstar	SP-01-D9	1

Electronics

Description	Manufacturer	Part Number	Quantity
Stranded wire, Solder, Flux	N/A	N/A	N/A
Banana clips (or another appropriate connector)	N/A	N/A	6
LED Driver	N/A	N/A	2

Assembly

LEDs

-Each LED requires:

- Milled as-1.50_2_v2.SLDPRT
- Spacer_with_wire_hole_as-.500_v2.SLDPRT
- LED_mount_as-1.50_v2.SLDPRT
- Retainer Ring
- 4-40 Bolts, 3/8 of an inch in length (3x)
- Thermal Paste

Cut three pairs of Positive and Ground wires of enough length (~ 1.0m), and expose the leads on each wire (~ 1/5 of an inch). Slip the pair of wires into the lateral hole of Spacer_with_wire_hole and push enough out of the top hole to slip into LED_mount (a few millimeters should show to solder onto the LED pads). Before adding LED-mount, add a retainer ring, and using a ¼-20 setscrew attach LED_mount_as-1.50_v2.SLDPRT. Attach Milled as-1.50_v2.SLDPRT to the bottom, the setscrew should be large enough to be able to hold everything in place. Finally, add thermal paste onto the top of LED_mount (a small drop will do), and attach the LED using the three 4-40 bolts. Solder the leads onto their respective Positive (+) and Ground (-) pads ensuring a proper connection with no crossing of leads. *Note: try and keep the soldering process as quick as possible to minimize heat transfer onto LED itself which may damage the diode.*

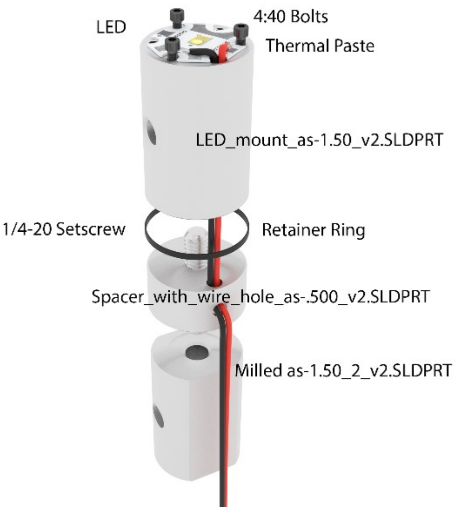

Repeat the above process for the other two LEDs, keeping track of which is which. Finally, you can add banana clips (or other appropriate clip) to the other end of the wires to make the attachment to the LED drivers easier.

LED Housings

-Each LED Housing requires:

- 3.0” Lens tube
- 0.5” Lens tube
- Bandpass filter of appropriate wavelength
- Retainer Ring (2x)
- Assembled LED of appropriate wavelength
- 25.4mm Bi-Convex lens

Remove the included retainer ring in both lens tubes using the spanner wrench from Thorlabs. Insert the bi-convex lens into the 3.0” lens tube and secure it using the retainer ring. Then insert the bandpass filter into the 0.5” lens tube and again, secure it in place using a retainer ring. Finally, add the assembled LED of appropriate wavelength matching the filter used and secure it using the retainer ring around the LED assembly.

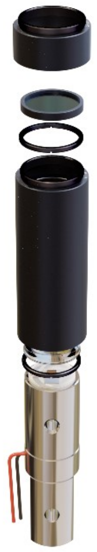

Dichroic Mirror Housing

For this step you will need the following:

- AT455DC Size: 26 * 38 mm
- 25 mm x 36 mm Longpass Dichroic Mirror, 550 nm Cutoff
- 30 mm Cage Cube with Dichroic Filter MountLED_mount_as-1.50_v2.SLDPRT
- Cage Cube Connector for Compact 30 mm Cage Cubes Thermal Paste

Unscrew the two screws to open up the housing of the mirror. Attach the dichroic mirrors as shown below ensuring proper alignment and positioning.

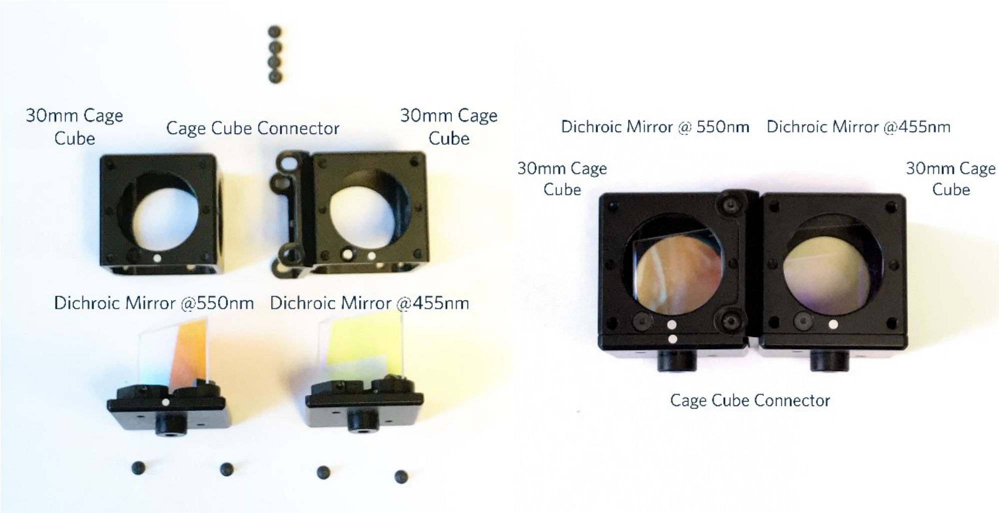

Finally, insert the last bi-convex lens into the 2.0” lens tube at a distance of ~30mm from where you insert it, and secure it using the retainer ring. Then add the liquid light guide adapter to the end and attach the light guide securing it by tightening the setscrew on the side. Assemble all the LEDs onto the cage housings ensuring proper placement as shown below. Then you can place it onto the 3D printed base.

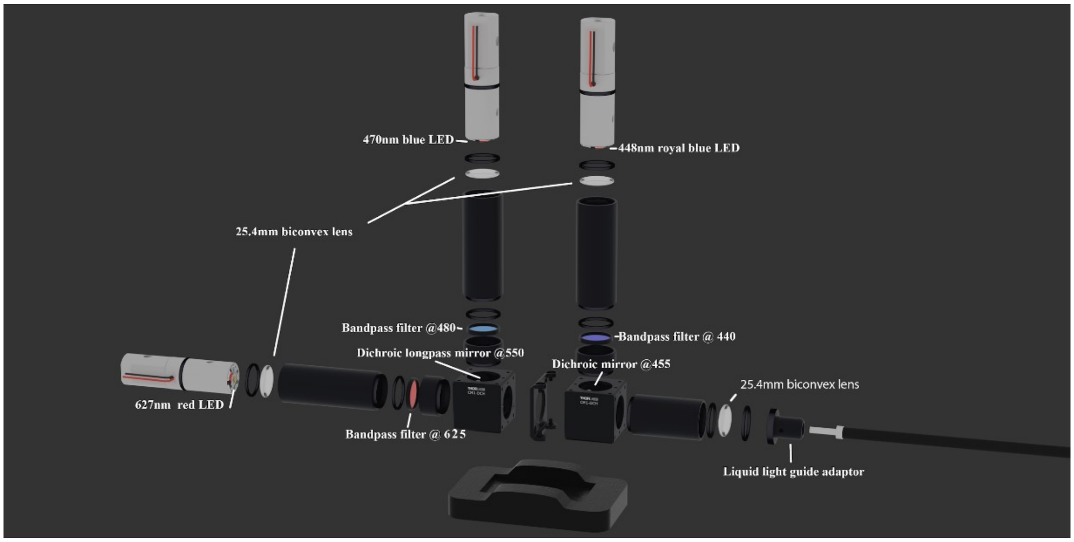

Electronics

Main Cage Wiring

Connections

The IR beam breakers, piezo buzzer, and vibrating motor are attached to our electronics box using JST connectors with either 3 or 2 positions with pins 2.5mm apart. The RFID tag reader is attached using an SD80LP plug. The servo motor is attached using the provided cable socket provided by Hitec., and the water solenoid is attached using a PTPM plug with 2 positions at a pitch of 2.5mm.

Electronic Box and Extension Breakout

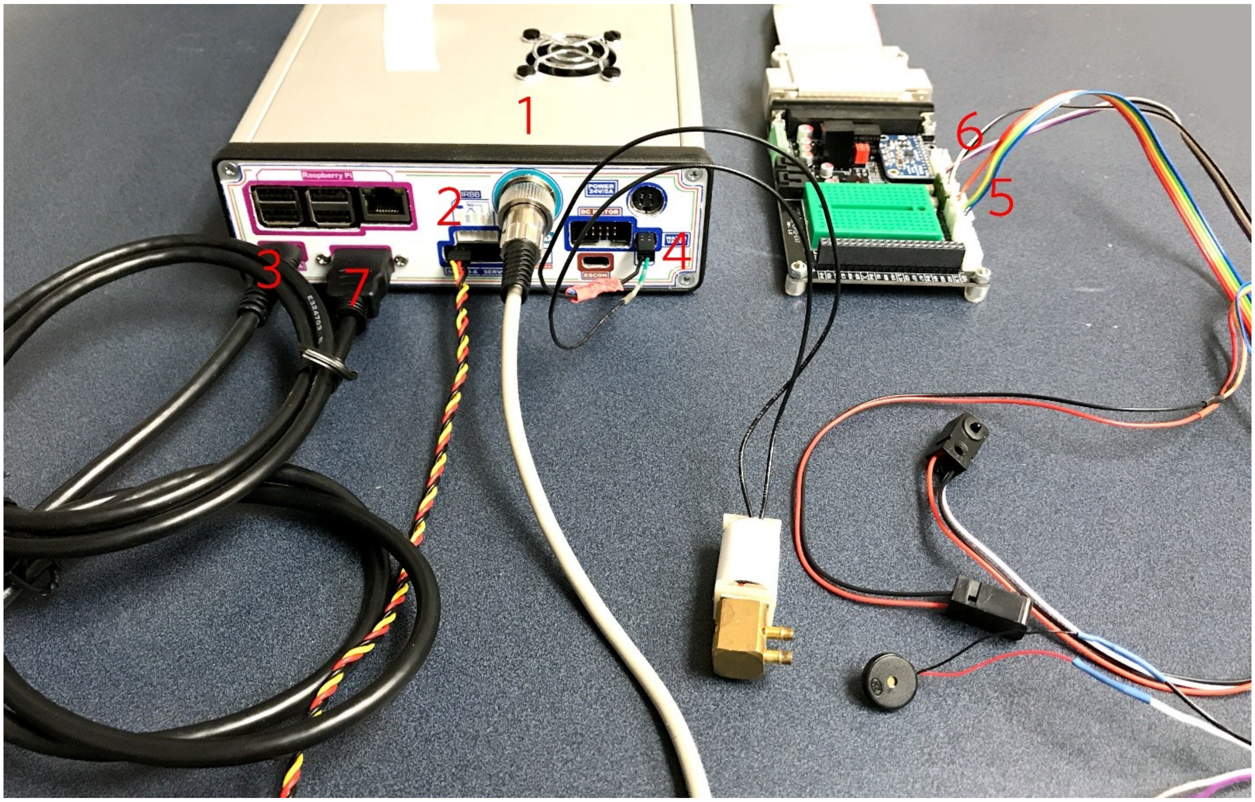

Our electronics box is labeled as such.

1) To RFID tag reader underneath head-fixing chamber
2) Servo-A: Servo motor for head-fixing
3) Mini-HDMI to Ribbon Cable for brain recordings
4) Water solenoid cables
5) IR beam breakers
6) Piezo Buzzer and Vibrating Motor
7) HDMI to monitor with USB ports above for mice/keyboards

Auto-Weighing Tunnel

The weighing tunnel is wired up alongside the second RFID tag reader. Using a small breadboard with a T-Cobbler attached to the a Raspberry Pi, attach the loadcell amplifier and RFID wires to their appropriate locations.

Behaviour Cameras

Triggered Recordings

Each behaviour camera will have their own Raspberry Pi with their ribbon cables attached. The triggering of recordings is done by the main head-fixing Raspberry Pi which sends a UDP network trigger.
