# Supplementary material for: Automated task training and longitudinal monitoring of mouse mesoscale cortical circuits using home cages
Source: eLife. 2020 May 15;9:e55964. doi: 10.7554/eLife.55964 (PMC7332290; doi:10.7554/eLife.55964)
Supplement: Supplementary file 2. [file elife-55964-supp2.zip › CAD_current_cage/LED_Parts/LED_mount_as-1.50_v2.PDF]

ITEM #	QTY	PART NUMBER	ASSY	DESCRIPTION	MATERIAL
1	1	STOCK			Aluminum

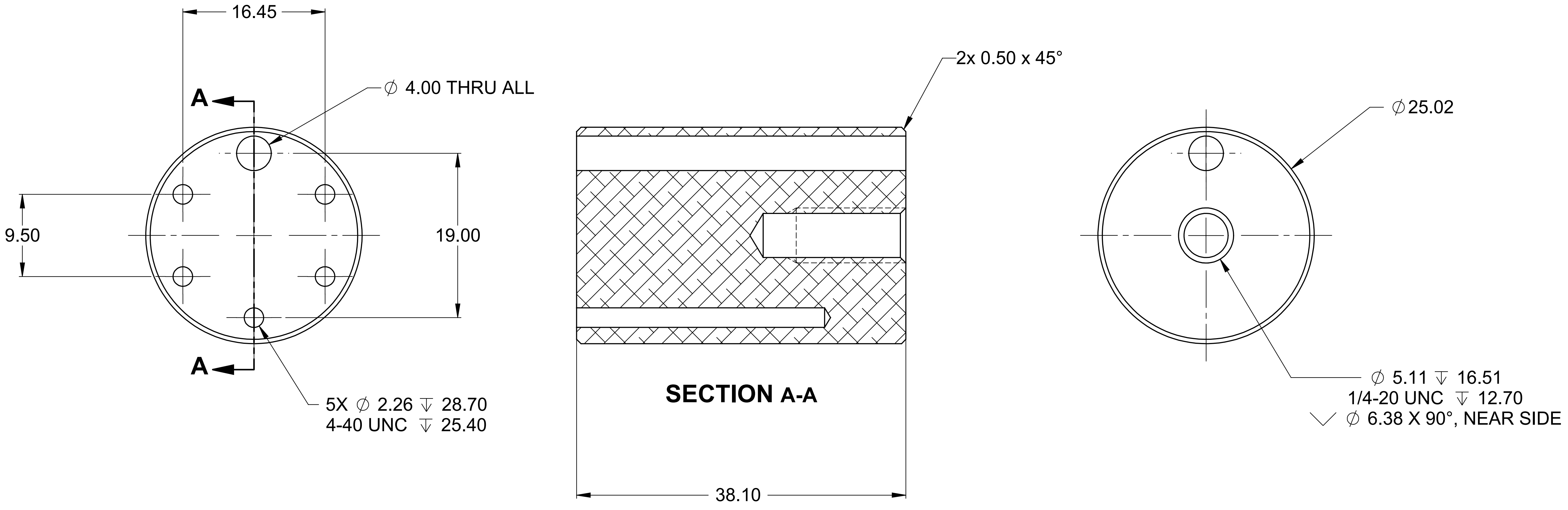

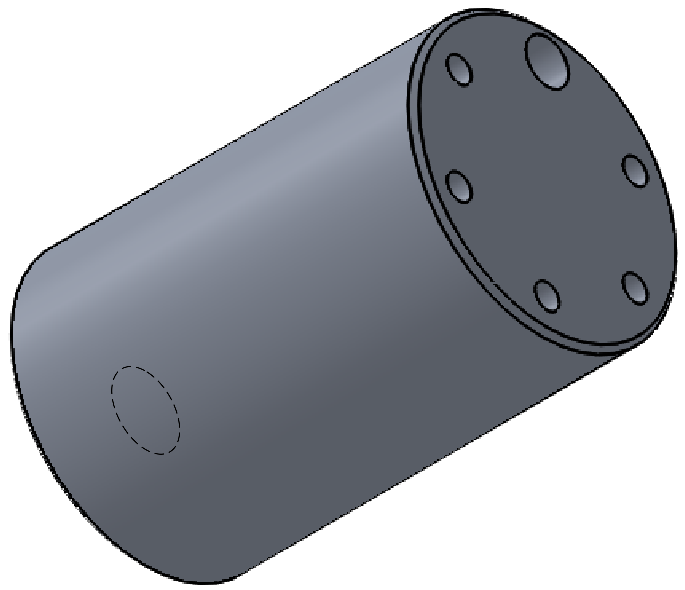

DRAWN F.L.	DATE Jan 24, 2017	DFTG APPVL		
MECH ENGR	ELEC ENGR	CIVIL ENGR	PHYSICS	ENGRG APPVL

REV	DESCRIPTION	DATE	DRAWN	APPVL

Drawing Name

LED_mount_as-1.50_v2

Project Name

Mounts and Spacers II

FINISH

METRIC DRAWING
UNITS: mm

THIRD ANGLE PROJ.

UNLESS OTHERWISE NOTED, ALL DIMENSIONS ARE IN MILLIMETERS
PERMISSABLE DIMENSIONAL DEVIATION:

TOLERANCES: DECIMALS	ANGLES	SURFACE
.X ± 0.1<br>.XX ± 0.05<br>.XXX ±	±	√

SIZE B | W.O. NO. M17-010 | ISSUE

SCALE 2:1 | SHEET 1 OF 1
