# Supplementary material for: Automated task training and longitudinal monitoring of mouse mesoscale cortical circuits using home cages
Source: eLife. 2020 May 15;9:e55964. doi: 10.7554/eLife.55964 (PMC7332290; doi:10.7554/eLife.55964)
Supplement: Supplementary file 2. [file elife-55964-supp2.zip › CAD_current_cage/test_bar/bar_with_hole.PDF]

ITEM #	QTY	PART NUMBER	ASSY	DESCRIPTION	MATERIAL
1	1	STOCK		1/8" Thick Bar	Steel

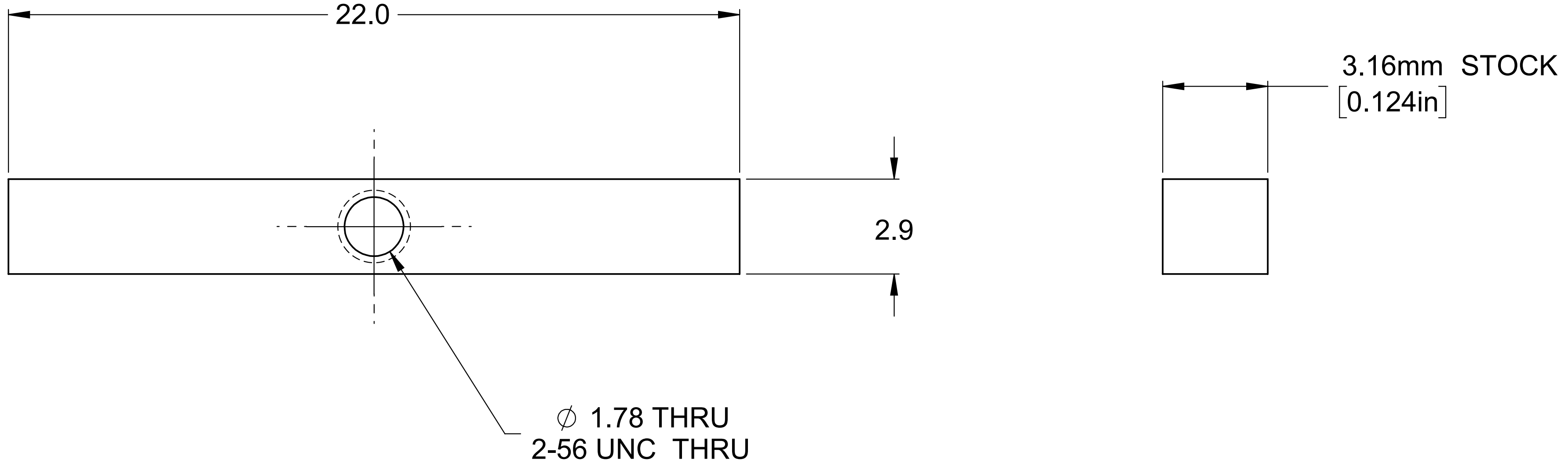

DRAWN	F.L.	DATE	Jan 30, 2017	DFTG APPVL
MECH ENGR	ELEC ENGR	CIVIL ENGR	PHYSICS	ENGRG APPVL

REV	DESCRIPTION	DATE	DRAWN	APPVL

Drawing Name

Bar

Project Name

Bar and Handle

FINISH

METRIC DRAWING
UNITS: mm

THIRD ANGLE PROJ.

UNLESS OTHERWISE NOTED, ALL DIMENSIONS ARE IN MILLIMETERS
PERMISSABLE DIMENSIONAL DEVIATION:

TOLERANCES: DECIMALS / ANGLES / SURFACE

.X ± 0.1
.XX ± 0.05
.XXX ±

±

√

SIZE B | W.O. NO. M17-012 | ISSUE

SCALE 5:1 | SHEET 1 OF 1
