# Supplementary material for: Automated task training and longitudinal monitoring of mouse mesoscale cortical circuits using home cages
Source: eLife. 2020 May 15;9:e55964. doi: 10.7554/eLife.55964 (PMC7332290; doi:10.7554/eLife.55964)
Supplement: Supplementary file 2. [file elife-55964-supp2.zip › CAD_current_cage/test_bar/handle_for_bar.PDF]

ITEM #	QTY	PART NUMBER	ASSY	DESCRIPTION	MATERIAL
1	1	STOCK			Steel

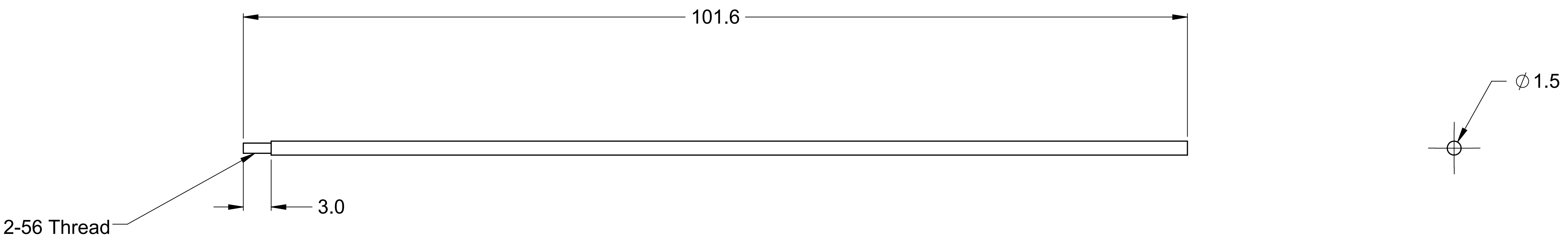

DRAWN F.L.	DATE Jan 30, 2017	DFTG APPVL		
MECH ENGR	ELEC ENGR	CIVIL ENGR	PHYSICS	ENGRG APPVL

REV	DESCRIPTION	DATE	DRAWN	APPVL

Drawing Name

Handle

Project Name

Bar and Handle

FINISH

METRIC DRAWING UNITS: mm

THIRD ANGLE PROJ.

UNLESS OTHERWISE NOTED, ALL DIMENSIONS ARE IN MILLIMETERS
PERMISSABLE DIMENSIONAL DEVIATION:

TOLERANCES: DECIMALS	ANGLES	SURFACE
.X ± 0.1<br>.XX ± 0.05<br>.XXX ±	±	√

SIZE	W.O. NO.	ISSUE
B	M17-012	

SCALE	SHEET	OF
2:1	1	1
