# Supplementary material for: Automated task training and longitudinal monitoring of mouse mesoscale cortical circuits using home cages
Source: eLife. 2020 May 15;9:e55964. doi: 10.7554/eLife.55964 (PMC7332290; doi:10.7554/eLife.55964)
Supplement: Supplementary file 3. [file elife-55964-supp3.zip › electronics_box_and_breakout/AHF_Homecage_control_LPTD_SchPcb v2a.pdf]

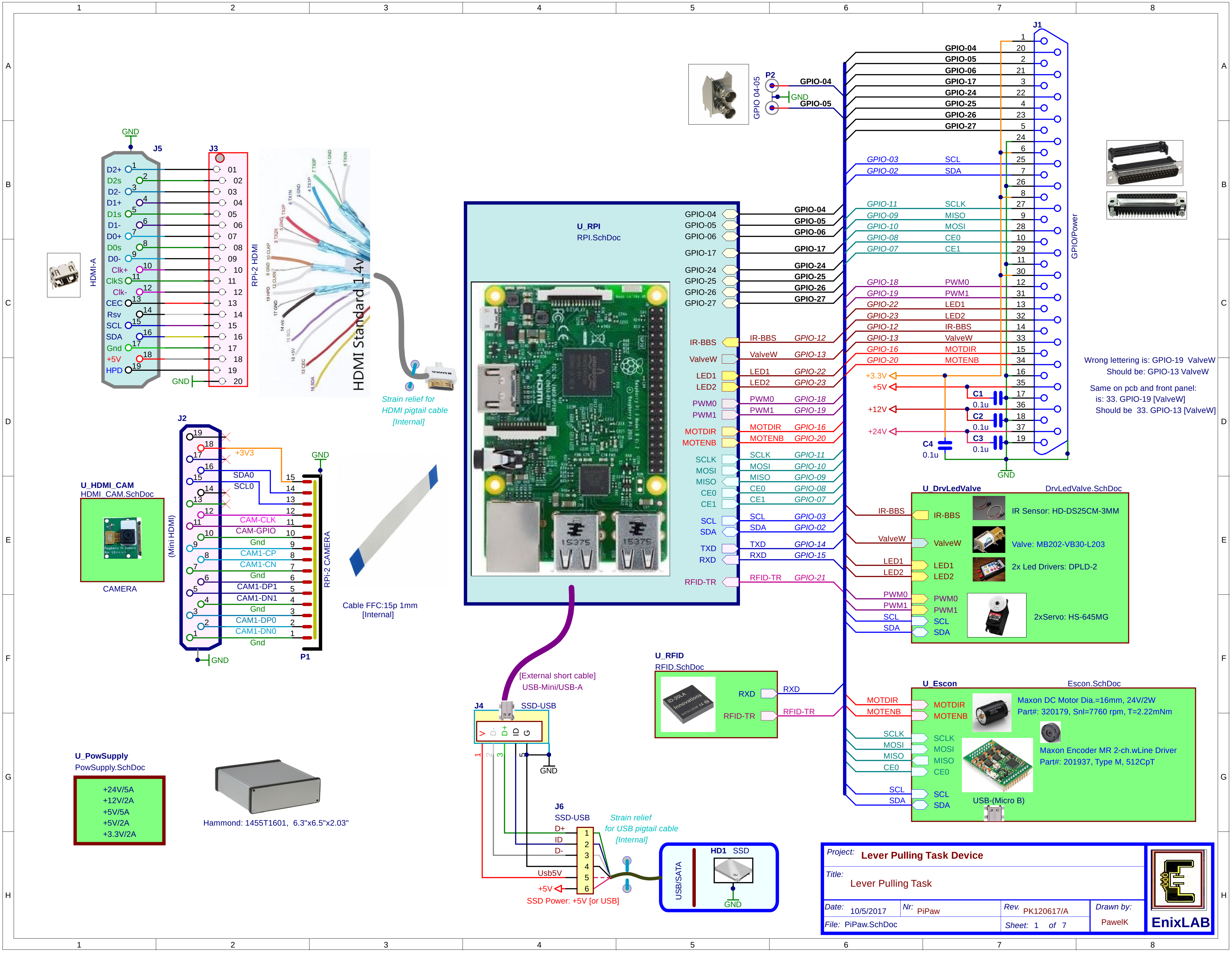

J1
GPIO/Power
GPIO-04
GPIO-05
GPIO-06
GPIO-17
GPIO-24
GPIO-25
GPIO-26
GPIO-27
GPIO-03 SCL
GPIO-02 SDA
GPIO-11 SCLK
GPIO-09 MISO
GPIO-10 MOSI
GPIO-08 CE0
GPIO-07 CE1
GPIO-18 PWM0
GPIO-19 PWM1
GPIO-22 LED1
GPIO-23 LED2
GPIO-12 IR-BBS
GPIO-13 ValveW
GPIO-16 MOTDIR
GPIO-20 MOTENB
+3.3V
+5V
+12V
+24V
C1 0.1u
C2 0.1u
C3 0.1u
C4 0.1u
GND
P2
GPIO 04-05
Wrong lettering is: GPIO-19 ValveW
Should be: GPIO-13 ValveW
Same on pcb and front panel:
is: 33. GPIO-19 [ValveW]
Should be 33. GPIO-13 [ValveW]
J5
J3
HDMI-A
RPi-2 HDMI
D2+
D2s
D2-
D1+
D1s
D1-
D0+
D0s
D0-
Clk+
ClkS
Clk-
CEC
Rsv
SCL
SDA
Gnd
+5V
HPD
HDMI Standard 1.4v
Strain relief for
HDMI pigtail cable
[Internal]
U_RPI
RPI.SchDoc
IR-BBS
ValveW
LED1
LED2
PWM0
PWM1
MOTDIR
MOTENB
SCLK
MOSI
MISO
CE0
CE1
SCL
SDA
TXD
RXD
RFID-TR
GPIO-14
GPIO-15
GPIO-21
J2
(Mini HDMI)
RPi-2 CAMERA
P1
+3V3
SDA0
SCL0
CAM-CLK
CAM-GPIO
CAM1-CP
CAM1-CN
CAM1-DP1
CAM1-DN1
CAM1-DP0
CAM1-DN0
U_HDMI_CAM
HDMI_CAM.SchDoc
CAMERA
Cable FFC:15p 1mm
[Internal]
U_DrvLedValve
DrvLedValve.SchDoc
IR Sensor: HD-DS25CM-3MM
Valve: MB202-VB30-L203
2x Led Drivers: DPLD-2
2xServo: HS-645MG
U_RFID
RFID.SchDoc
U_Escon
Escon.SchDoc
Maxon DC Motor Dia.=16mm, 24V/2W
Part#: 320179, Snl=7760 rpm, T=2.22mNm
Maxon Encoder MR 2-ch.wLine Driver
Part#: 201937, Type M, 512CpT
USB-(Micro B)
[External short cable]
USB-Mini/USB-A
J4
SSD-USB
J6
SSD-USB
D+
ID
D-
Usb5V
Strain relief
for USB pigtail cable
[Internal]
HD1 SSD
USB/SATA
SSD Power: +5V [or USB]
U_PowSupply
PowSupply.SchDoc
+24V/5A
+12V/2A
+5V/5A
+5V/2A
+3.3V/2A
Hammond: 1455T1601, 6.3"x6.5"x2.03"
Project: Lever Pulling Task Device
Title: Lever Pulling Task
Date: 10/5/2017
Nr: PiPaw
Rev. PK120617/A
Drawn by: PawelK
File: PiPaw.SchDoc
Sheet: 1 of 7
EnixLAB

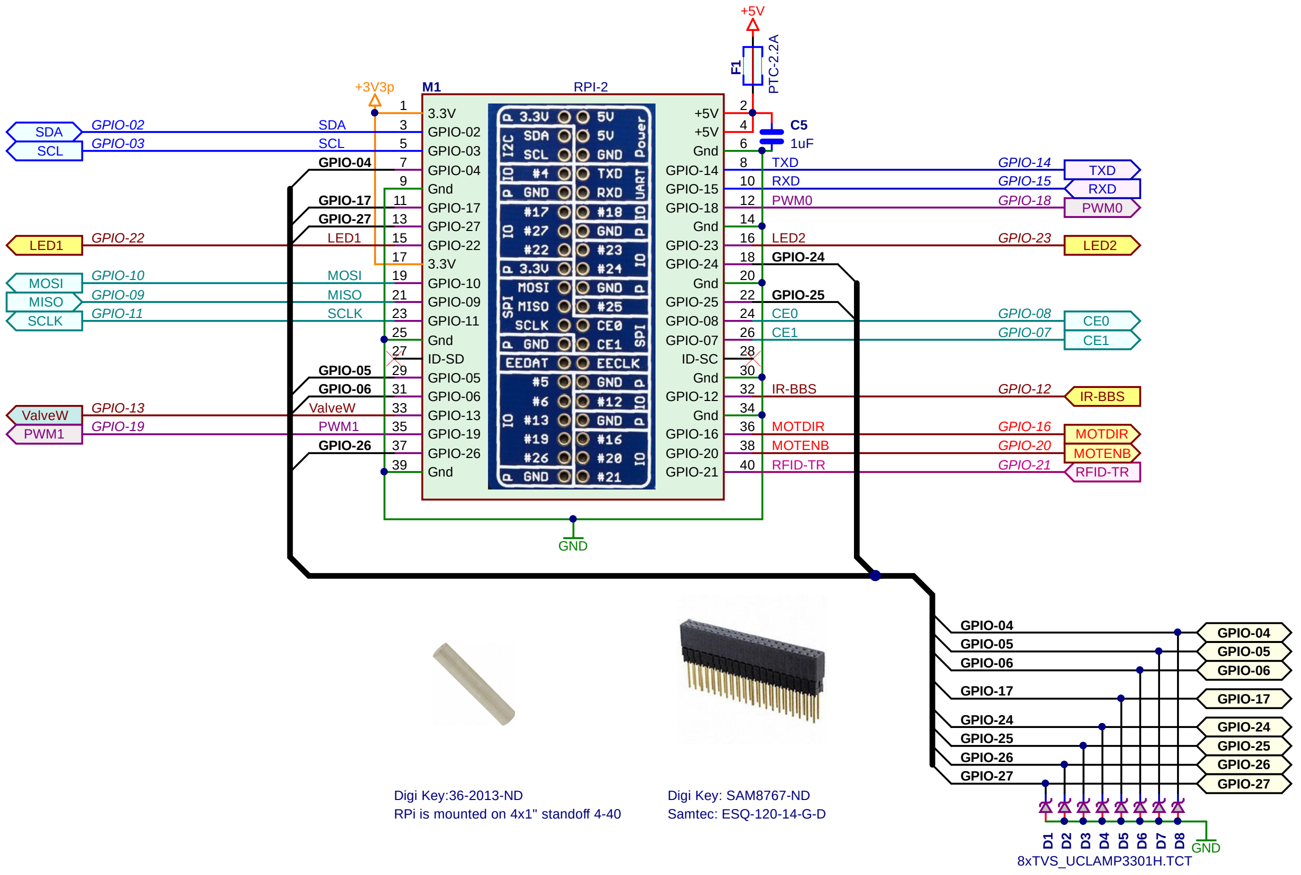

Project: **Lever Pulling Task Device**

Title: Raspberry PI-2 GPIO

Date: 9/2/2017	Nr: PiPaw	Rev. PK120617/A	Drawn by: PawelK
File: RPI.SchDoc		Sheet: 2 of 7	

EnixLAB

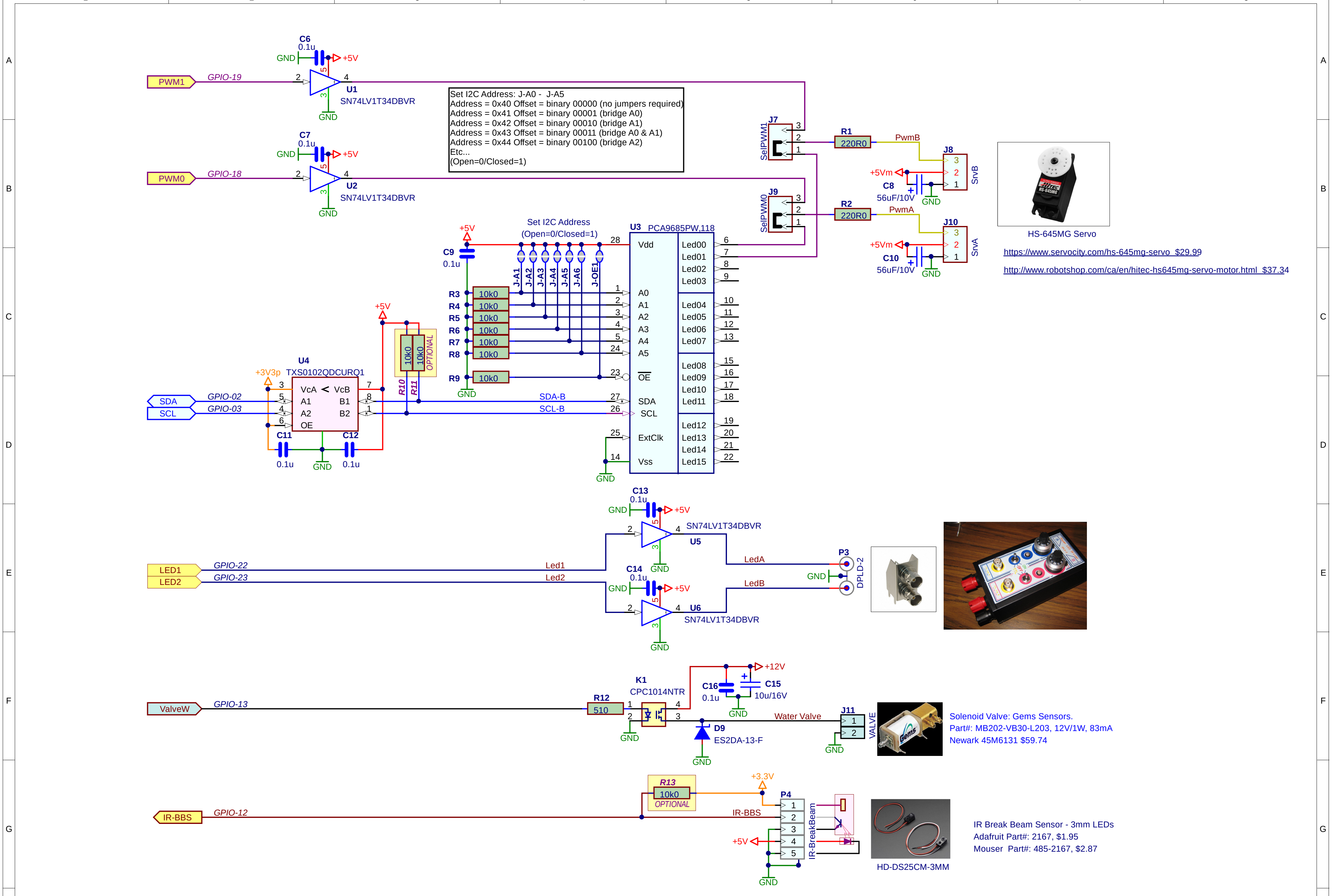

Project:	Lever Pulling Task Device
Title:	Drivers: Servo, Led's, Valve & IR Breaker
Date: 9/2/2017	Nr: PiPaw
Rev. PK120617/A	Drawn by: PawelK
File: DrvLedValve.SchDoc	Sheet: 3 of 7

EnixLAB

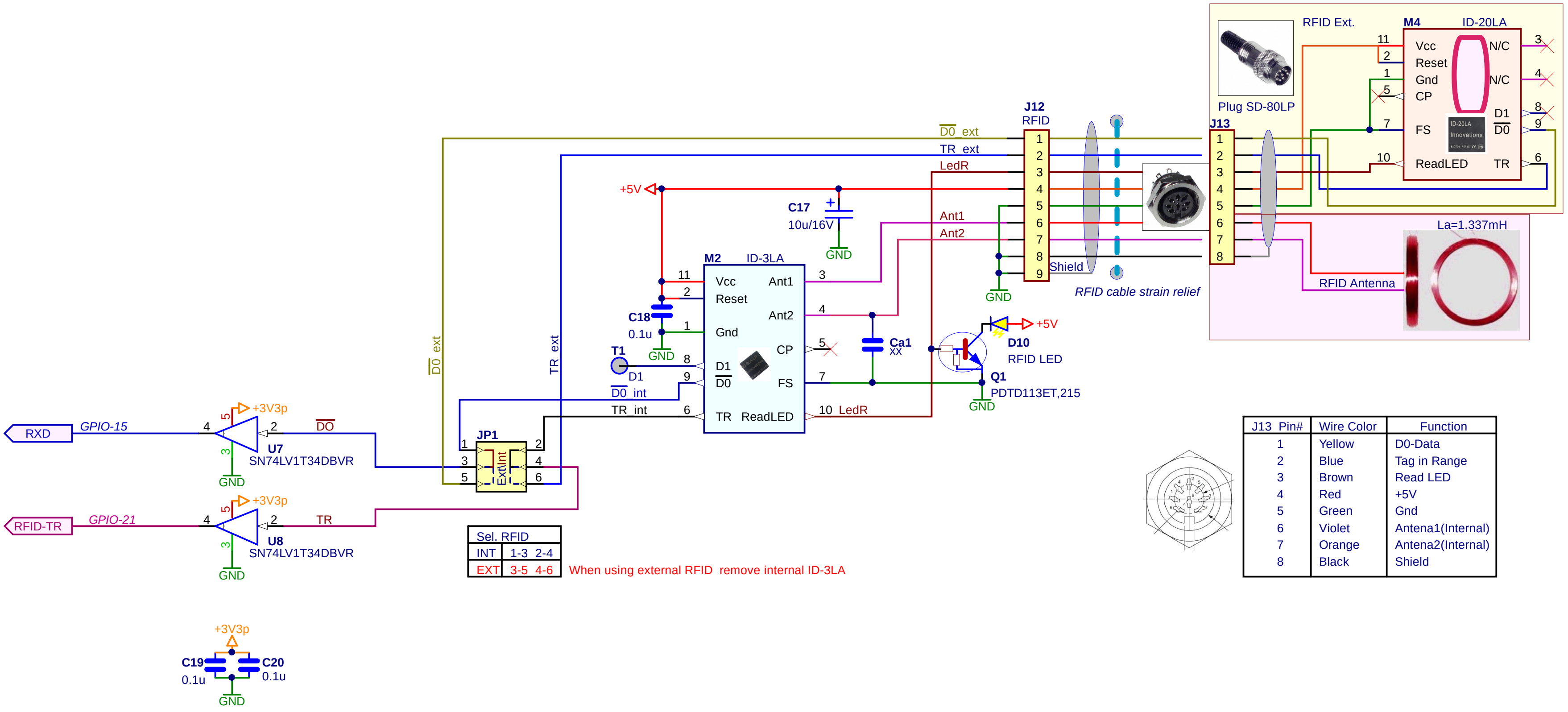

J13 Pin#	Wire Color	Function
1	Yellow	D0-Data
2	Blue	Tag in Range
3	Brown	Read LED
4	Red	+5V
5	Green	Gnd
6	Violet	Antena1(Internal)
7	Orange	Antena2(Internal)
8	Black	Shield

Project: **Lever Pulling Task Device**

Title: RFID Inernal/External

Date: 8/29/2017 | Nr: PiPaw | Rev. PK120617/A | Drawn by: PawelK

File: RFID.SchDoc | Sheet: 4 of 7

EnixLAB

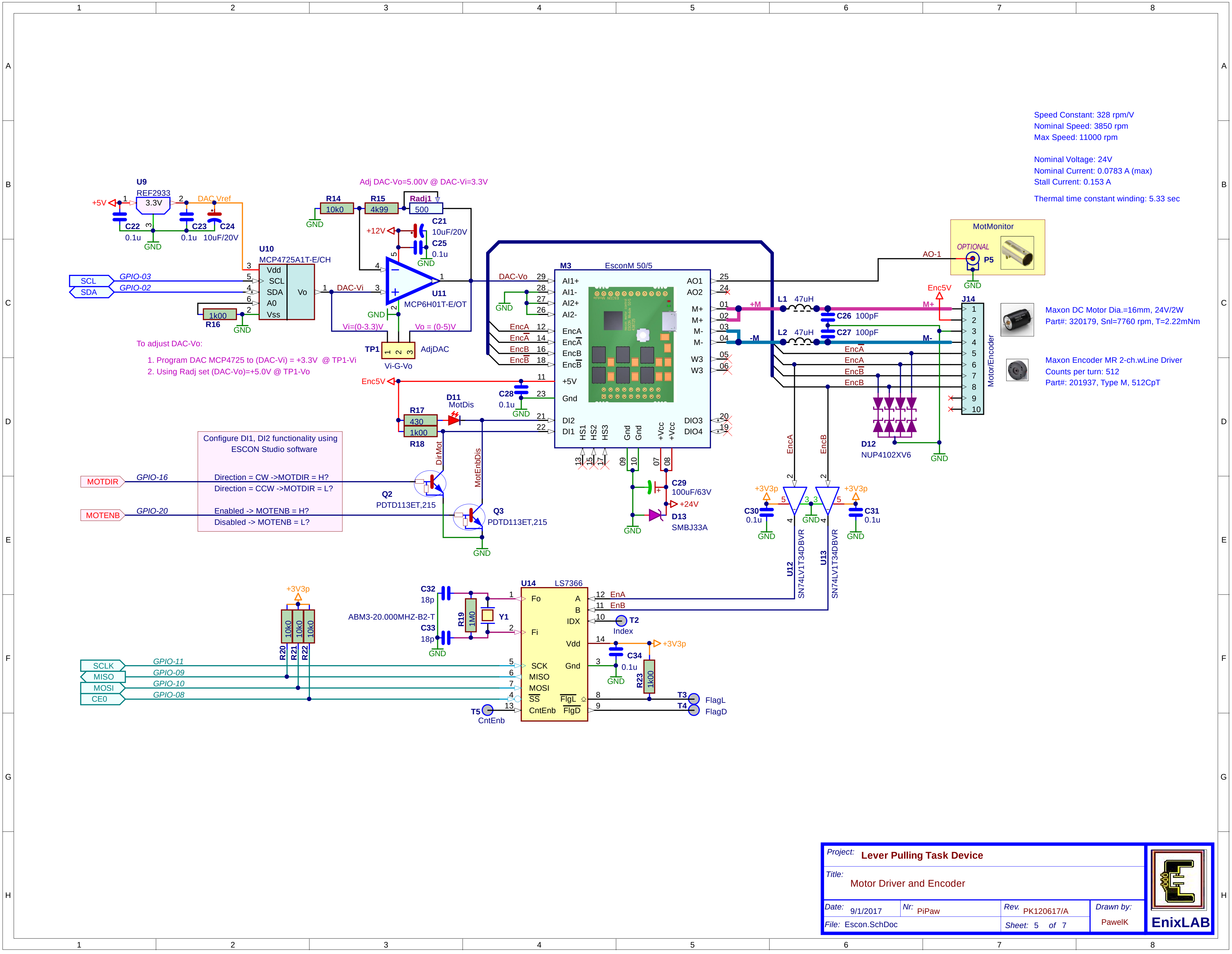
Speed Constant: 328 rpm/V
Nominal Speed: 3850 rpm
Max Speed: 11000 rpm
Nominal Voltage: 24V
Nominal Current: 0.0783 A (max)
Stall Current: 0.153 A
Thermal time constant winding: 5.33 sec
Adj DAC-Vo=5.00V @ DAC-Vi=3.3V
U9 REF2933 3.3V
DAC Vref
C22 0.1u
C23 0.1u
C24 10uF/20V
U10 MCP4725A1T-E/CH
SCL GPIO-03
SDA GPIO-02
R16 1k00
R14 10k0
R15 4k99
Radj1 500
C21 10uF/20V
C25 0.1u
+12V
DAC-Vi
DAC-Vo
U11 MCP6H01T-E/OT
Vi=(0-3.3V)
Vo = (0-5)V
TP1 AdjDAC Vi-G-Vo
To adjust DAC-Vo:
1. Program DAC MCP4725 to (DAC-Vi) = +3.3V @ TP1-Vi
2. Using Radj set (DAC-Vo)=+5.0V @ TP1-Vo
M3 EsconM 50/5
MotMonitor
OPTIONAL
AO-1
P5
Enc5V
J14 Motor/Encoder
L1 47uH
L2 47uH
C26 100pF
C27 100pF
+M
-M
Maxon DC Motor Dia.=16mm, 24V/2W
Part#: 320179, Snl=7760 rpm, T=2.22mNm
Maxon Encoder MR 2-ch.wLine Driver
Counts per turn: 512
Part#: 201937, Type M, 512CpT
D12 NUP4102XV6
C28 0.1u
R17 430
R18 1k00
D11 MotDis
DirMot
MotEnbDis
Configure DI1, DI2 functionality using ESCON Studio software
MOTDIR GPIO-16
Direction = CW ->MOTDIR = H?
Direction = CCW ->MOTDIR = L?
MOTENB GPIO-20
Enabled -> MOTENB = H?
Disabled -> MOTENB = L?
Q2 PDTD113ET,215
Q3 PDTD113ET,215
C29 100uF/63V
+24V
D13 SMBJ33A
EncA
EncB
C30 0.1u
C31 0.1u
+3V3p
U12 SN74LV1T34DBVR
U13 SN74LV1T34DBVR
U14 LS7366
C32 18p
C33 18p
R19 1M0
Y1 ABM3-20.000MHZ-B2-T
EnA
EnB
T2 Index
C34 0.1u
R23 1k00
T3 FlagL
T4 FlagD
T5 CntEnb
R20 10k0
R21 10k0
R22 10k0
SCLK GPIO-11
MISO GPIO-09
MOSI GPIO-10
CE0 GPIO-08
GND
Project: Lever Pulling Task Device
Title: Motor Driver and Encoder
Date: 9/1/2017
Nr: PiPaw
Rev. PK120617/A
Drawn by: PawelK
File: Escon.SchDoc
Sheet: 5 of 7
EnixLAB

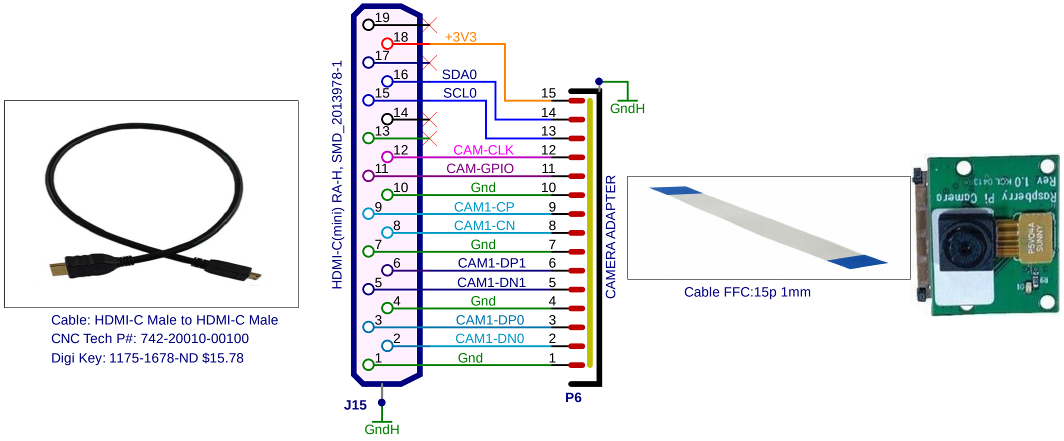

Project:	**Lever Pulling Task Device**
Title:	HDMI, Camera Adapter
Date: 8/13/2017	*Nr:* PiPaw
Rev. PK120617/A	*Drawn by:* PawelK
File: HDMI_CAM.SchDoc	*Sheet:* 6 of 7

EnixLAB

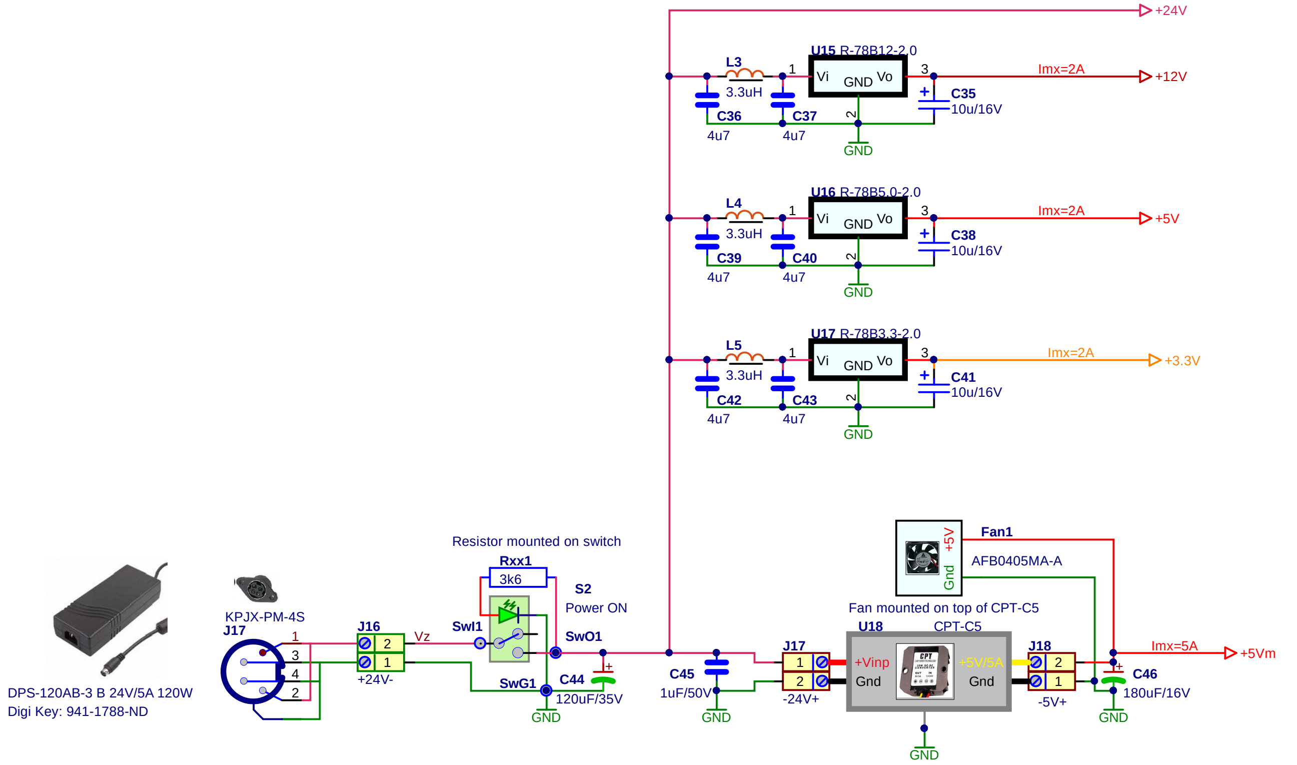

Project: **Lever Pulling Task Device**

Title: Power Supply and Regulators +24V, +12V, 2x +5V, +3.3V

Date: 8/20/2017	*Nr:* PiPaw	*Rev.* PK120617/A	*Drawn by:* PawelK
File: PowSupply.SchDoc		*Sheet:* 7 of 7	

EnixLAB

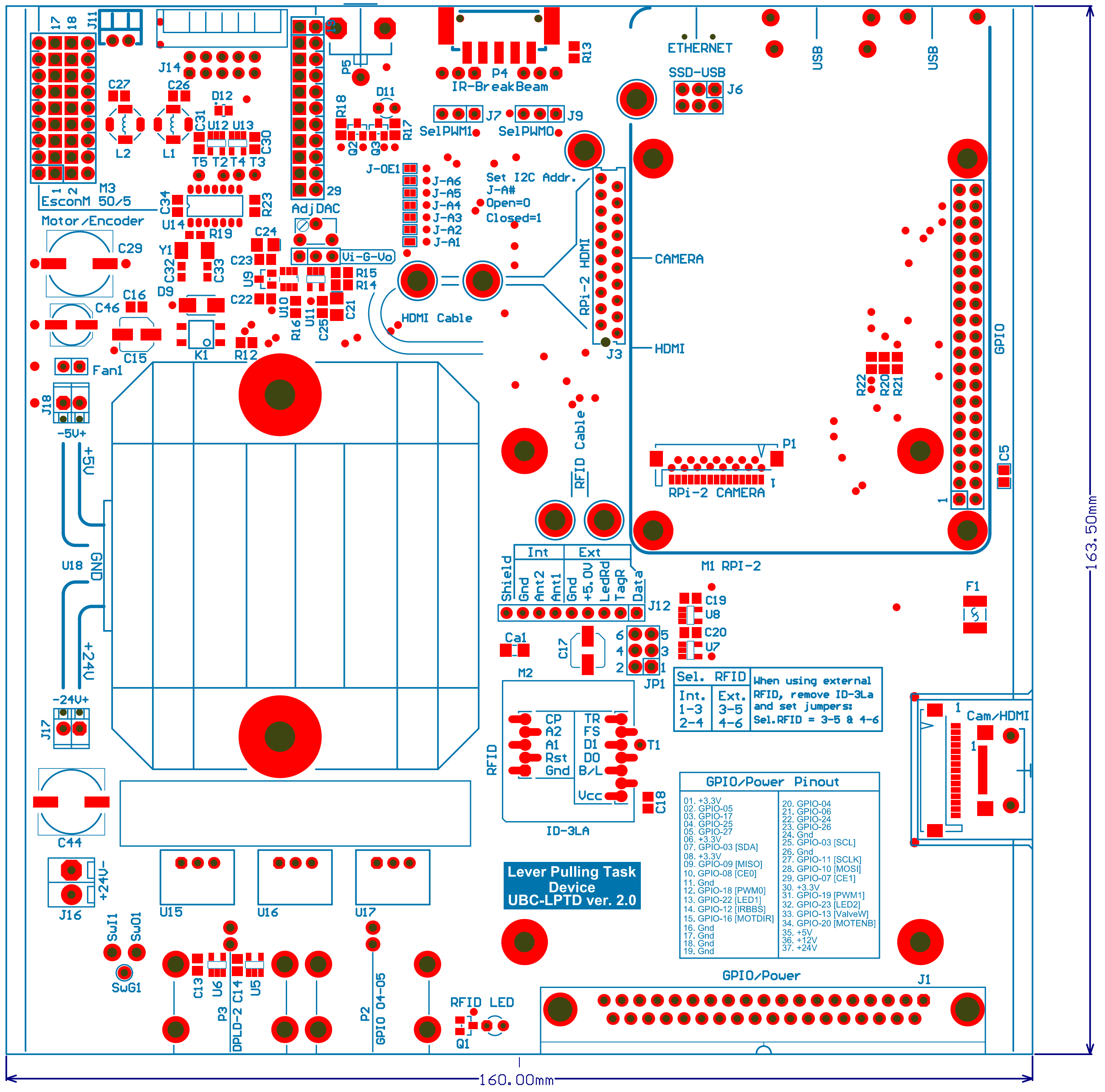

ETHERNET
USB
USB
SSD-USB
J6
IR-BreakBeam
P4
J7
J9
SelPWM1
SelPWM0
Set I2C Addr.
J-A#
Open=0
Closed=1
J-OE1
J-A6
J-A5
J-A4
J-A3
J-A2
J-A1
CAMERA
HDMI
RPi-2 HDMI
J3
HDMI Cable
RFID Cable
GPIO
RPi-2 CAMERA
P1
M1 RPI-2
EsconM 50/5
Motor/Encoder
M3
J14
AdjDAC
Vi-G-Vo
Fan1
-5V+
+5V
GND
U18
+24V
-24V+
J17
J18
C44
J16
+24V-
SwG1
Sw1
Sw0
U15
U16
U17
P3
OPLD-2
P2
GPIO 04-05
RFID LED
Q1
Int
Ext
Shield
Gnd
Ant2
Ant1
Gnd
+5.0V
LedRd
TagR
Data
J12
JP1
Cal
M2
RFID
CP
A2
A1
Rst
Gnd
TR
FS
D1
D0
B/L
Vcc
T1
ID-3LA
Sel. RFID
Int.
1-3
2-4
Ext.
3-5
4-6
When using external
RFID, remove ID-3La
and set jumpers:
Sel.RFID = 3-5 & 4-6
Cam/HDMI
F1
Lever Pulling Task
Device
UBC-LPTD ver. 2.0
GPIO/Power Pinout
01. +3.3V
02. GPIO-05
03. GPIO-17
04. GPIO-25
05. GPIO-27
06. +3.3V
07. GPIO-03 [SDA]
08. +3.3V
09. GPIO-09 [MISO]
10. GPIO-08 [CE0]
11. Gnd
12. GPIO-18 [PWM0]
13. GPIO-22 [LED1]
14. GPIO-12 [IRBBS]
15. GPIO-16 [MOTDIR]
16. Gnd
17. Gnd
18. Gnd
19. Gnd
20. GPIO-04
21. GPIO-06
22. GPIO-24
23. GPIO-26
24. Gnd
25. GPIO-03 [SCL]
26. Gnd
27. GPIO-11 [SCLK]
28. GPIO-10 [MOSI]
29. GPIO-07 [CE1]
30. +3.3V
31. GPIO-19 [PWM1]
32. GPIO-23 [LED2]
33. GPIO-13 [ValveW]
34. GPIO-20 [MOTENB]
35. +5V
36. +12V
37. +24V
GPIO/Power
J1
163.50mm
160.00mm

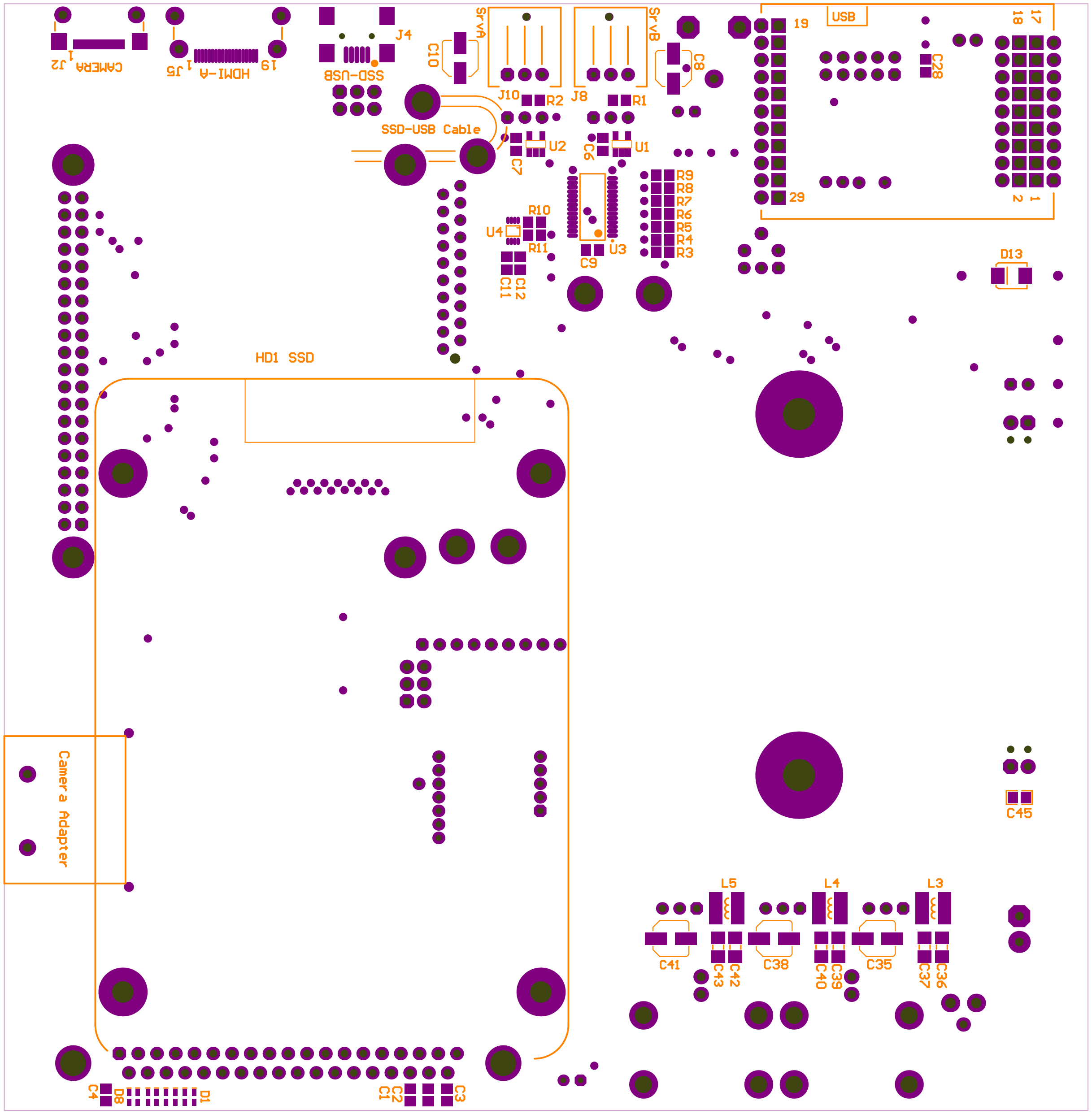

CAMERA J2
HDMI-A J5
SSD-USB
J4
C10
SrvA
J10
R2
J8
R1
SrvB
C8
USB
19
29
C28
SSD-USB Cable
U2
C7
C6
U1
R9
R8
R7
R6
R5
R4
R3
R10
U4
R11
C11
C12
C9
U3
D13
HD1 SSD
Camera Adapter
C45
L5
L4
L3
C41
C43
C42
C38
C40
C39
C35
C37
C36
C4
D8
D1
C1
C2
C3
