# Supplementary material for: Automated task training and longitudinal monitoring of mouse mesoscale cortical circuits using home cages
Source: eLife. 2020 May 15;9:e55964. doi: 10.7554/eLife.55964 (PMC7332290; doi:10.7554/eLife.55964)
Supplement: Supplementary file 3. [file elife-55964-supp3.zip › electronics_box_and_breakout/AHFboxFront.pdf]

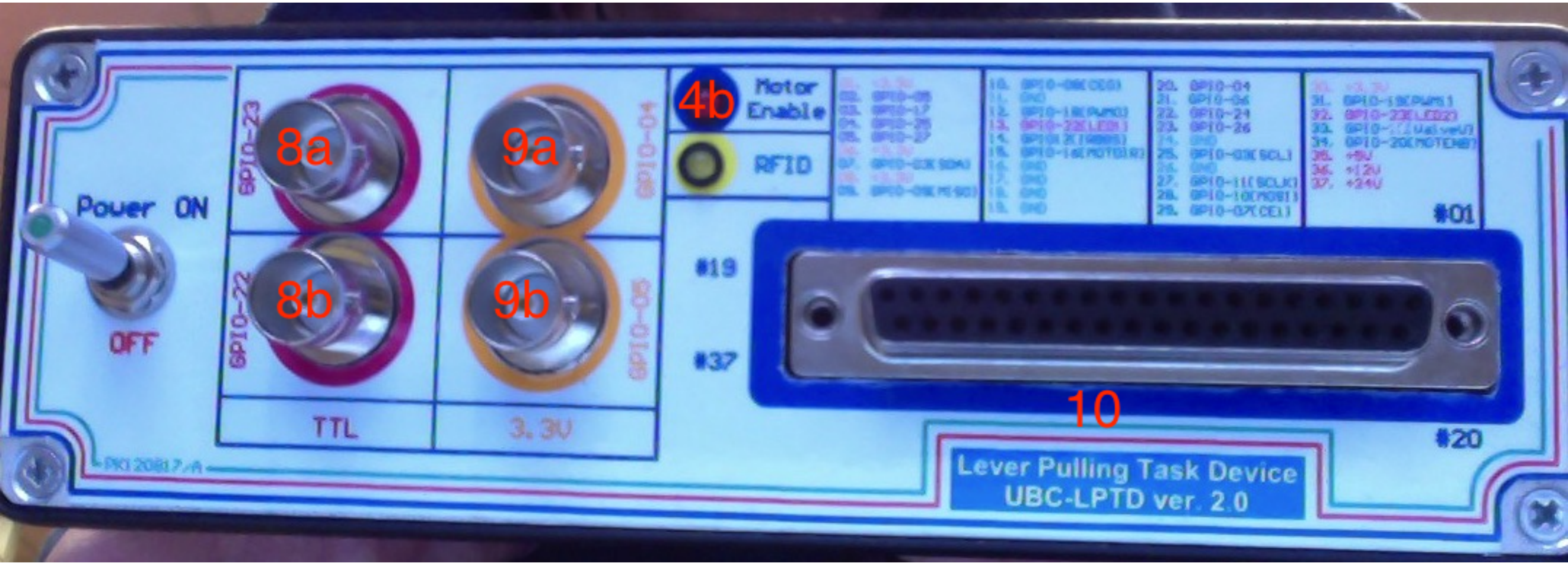

Power ON
OFF
GPIO-23
GPIO-22
8a
8b
9a
9b
GPIO-04
GPIO-05
TTL
3.3V
4b
Motor Enable
RFID
#19
#37
#01
#20
10
Lever Pulling Task Device
UBC-LPTD ver. 2.0
