# Supplementary material for: Automated task training and longitudinal monitoring of mouse mesoscale cortical circuits using home cages
Source: eLife. 2020 May 15;9:e55964. doi: 10.7554/eLife.55964 (PMC7332290; doi:10.7554/eLife.55964)
Supplement: Supplementary file 3. [file elife-55964-supp3.zip › electronics_box_and_breakout/Breakout_LPDT_Ext.pdf]

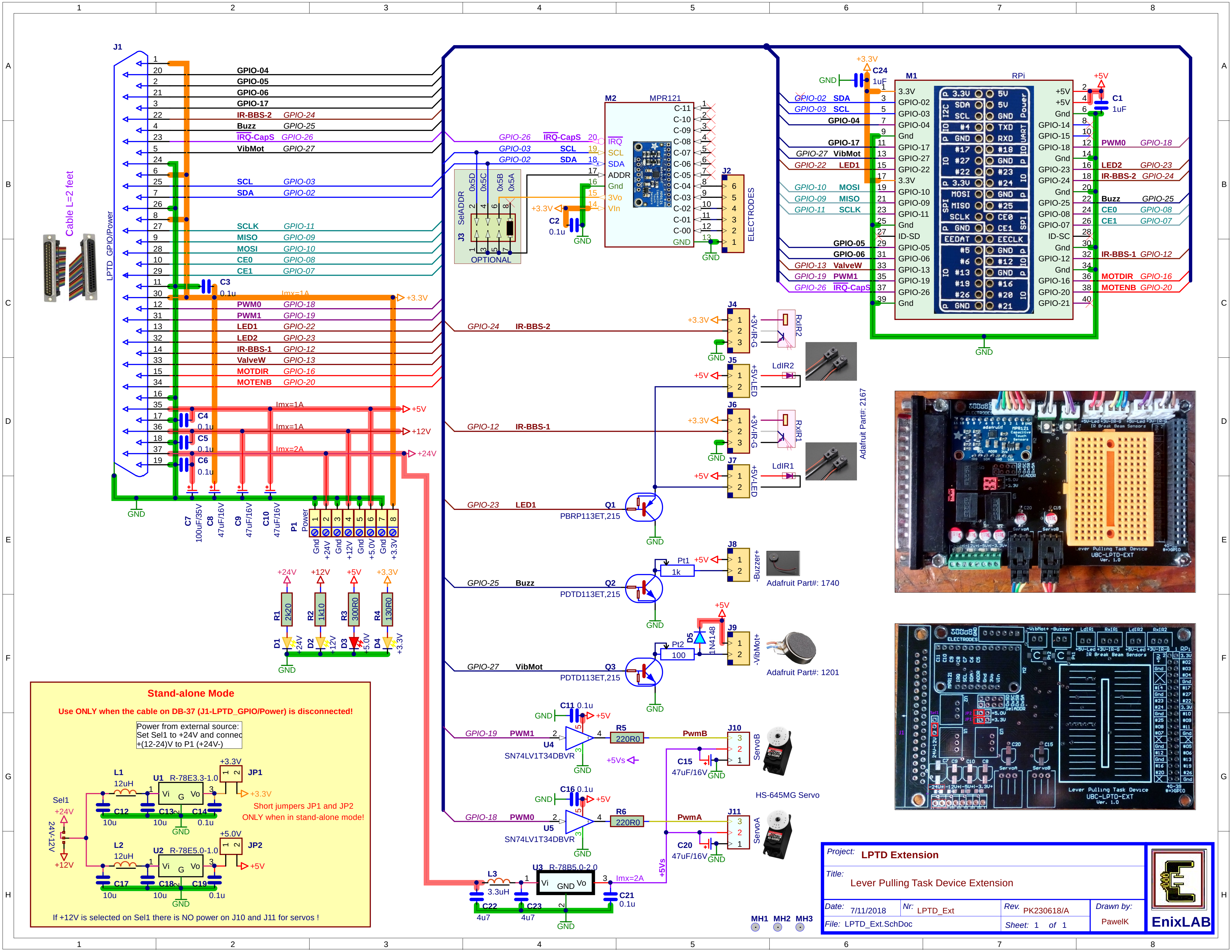

J1
Cable L=2 feet
LPTD GPIO/Power
GPIO-04
GPIO-05
GPIO-06
GPIO-17
IR-BBS-2 GPIO-24
Buzz GPIO-25
IRQ-CapS GPIO-26
VibMot GPIO-27
SCL GPIO-03
SDA GPIO-02
SCLK GPIO-11
MISO GPIO-09
MOSI GPIO-10
CE0 GPIO-08
CE1 GPIO-07
PWM0 GPIO-18
PWM1 GPIO-19
LED1 GPIO-22
LED2 GPIO-23
IR-BBS-1 GPIO-12
ValveW GPIO-13
MOTDIR GPIO-16
MOTENB GPIO-20
Imx=1A
Imx=2A
+3.3V
+5V
+12V
+24V
GND
C3 0.1u
C4 0.1u
C5 0.1u
C6 0.1u
C7 100uF/35V
C8 47uF/16V
C9 47uF/16V
C10 47uF/16V
P1 Power
Gnd +24V Gnd +12V Gnd +5.0V Gnd +3.3V
R1 2k20
R2 1k10
R3 300R0
R4 130R0
D1 D2 D3 D4
M2 MPR121
J3 SelADDR
0x5D 0x5C 0x5B 0x5A
OPTIONAL
C2 0.1u
J2 ELECTRODES
M1 RPi
C24 1uF
C1 1uF
J4 +3V-IR-G RxIR2
J5 +5V-LED LdIR2
J6 +3V-IR-G RxIR1
J7 +5V-LED LdIR1
Adafruit Part#: 2167
Q1 PBRP113ET,215
J8 -Buzzer+
Pt1 1k
Adafruit Part#: 1740
Q2 PDTD113ET,215
D5 1N4148
J9 -VibMot+
Pt2 100
Adafruit Part#: 1201
Q3 PDTD113ET,215
C11 0.1u
R5 220R0
PwmB
J10 ServoB
U4 SN74LV1T34DBVR
C15 47uF/16V
HS-645MG Servo
C16 0.1u
R6 220R0
PwmA
J11 ServoA
U5 SN74LV1T34DBVR
C20 47uF/16V
+5Vs
L3 3.3uH
U3 R-78B5.0-2.0
C21 0.1u
C22 4u7
C23 4u7
Stand-alone Mode
Use ONLY when the cable on DB-37 (J1-LPTD_GPIO/Power) is disconnected!
Power from external source:
Set Sel1 to +24V and connec
+(12-24)V to P1 (+24V-)
Sel1
L1 12uH
U1 R-78E3.3-1.0
JP1
C12 10u
C13 10u
C14 0.1u
Short jumpers JP1 and JP2
ONLY when in stand-alone mode!
L2 12uH
U2 R-78E5.0-1.0
JP2
C17 10u
C18 10u
C19 0.1u
If +12V is selected on Sel1 there is NO power on J10 and J11 for servos !
MH1 MH2 MH3
Project: LPTD Extension
Title: Lever Pulling Task Device Extension
Date: 7/11/2018
Nr: LPTD_Ext
Rev. PK230618/A
Drawn by: PawelK
File: LPTD_Ext.SchDoc
Sheet: 1 of 1
EnixLAB

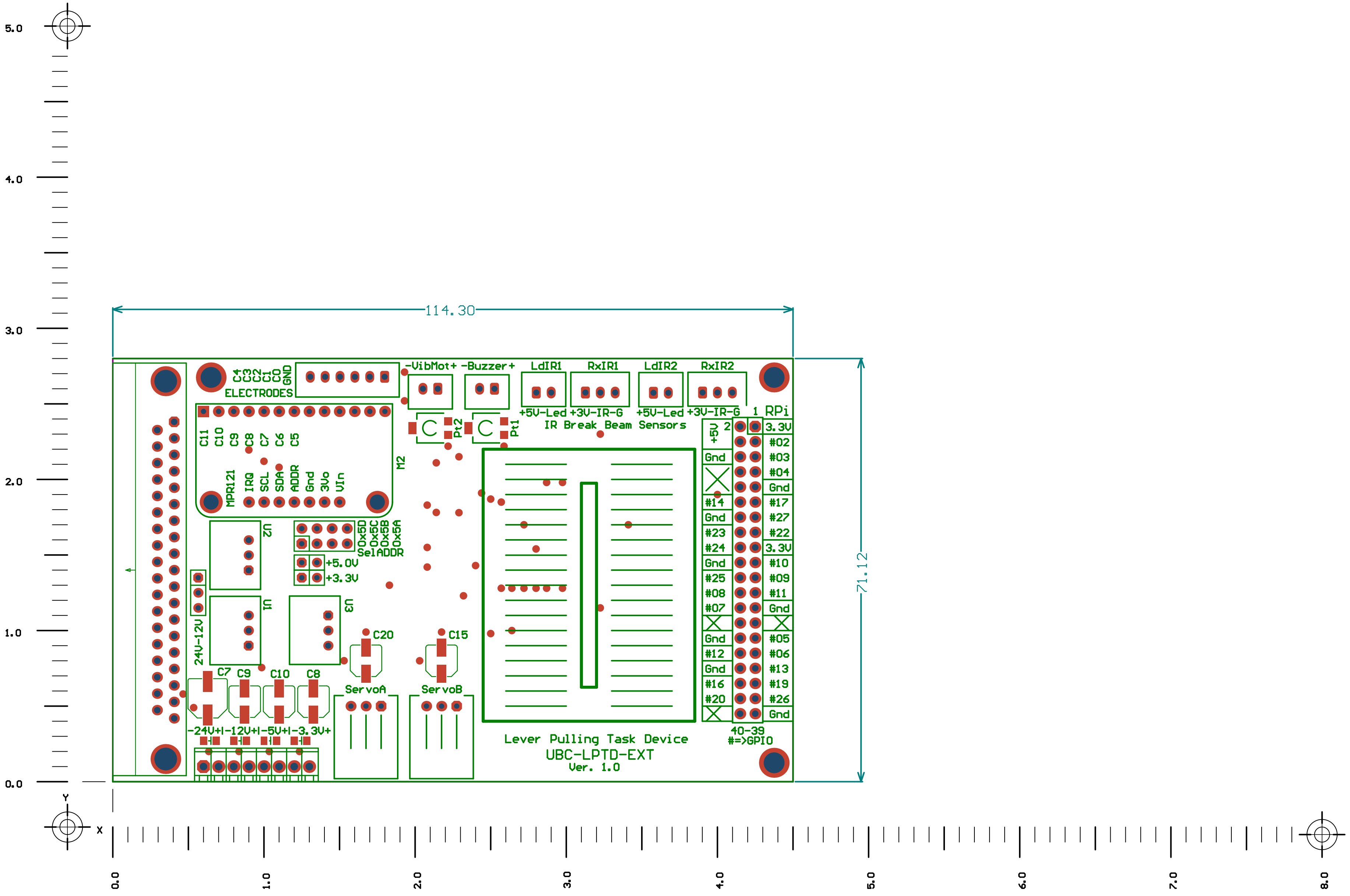

EnixLab

Designer: PawelK	Title: LPTD-Ext	Layer:	
Phone:			
e-Mail:	Part Nr: UBC-LPTD-Ext		Rev: PK230618/A
File: LPTD_Ext.PcbDoc		Date: 7/11/2018	SCALE: 1.07
