# Supplementary figures and images for: Automated task training and longitudinal monitoring of mouse mesoscale cortical circuits using home cages
Source: eLife. 2020 May 15;9:e55964. doi: 10.7554/eLife.55964 (PMC7332290; doi:10.7554/eLife.55964)

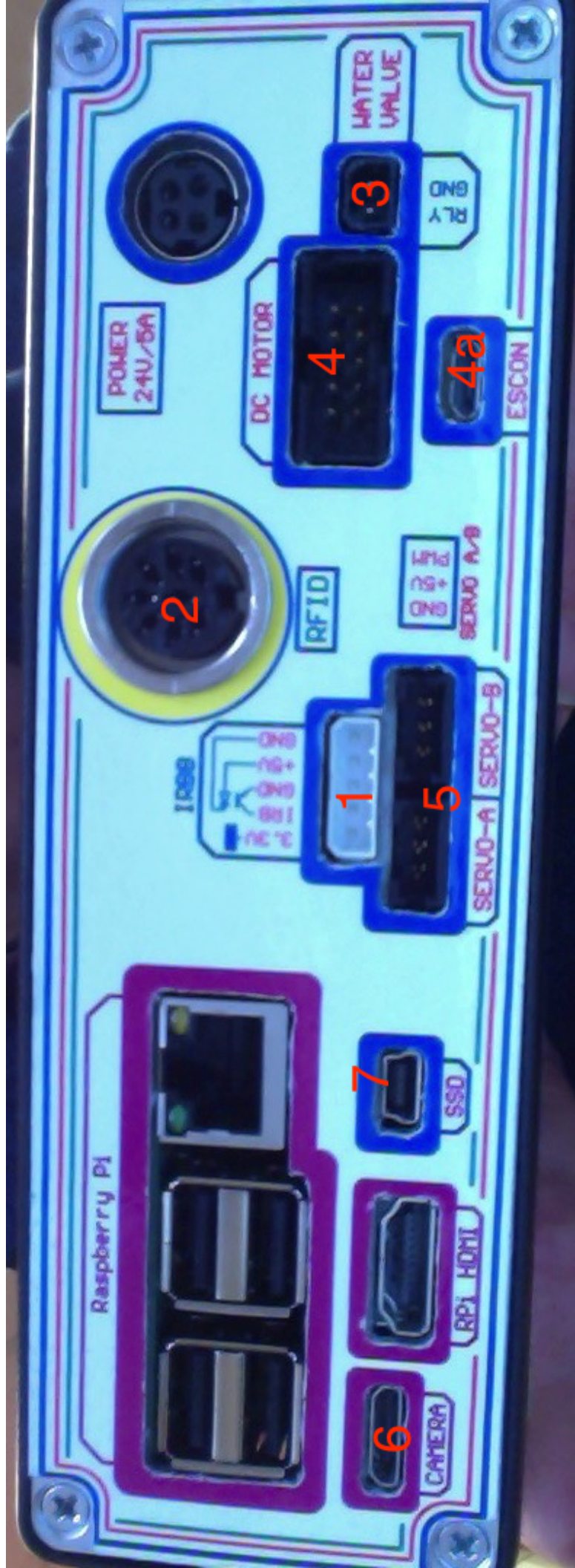

Supplement: Supplementary file 3. [file elife-55964-supp3.zip › electronics_box_and_breakout/AHFboxRear.pdf]
