# Supplementary material for: Automated task training and longitudinal monitoring of mouse mesoscale cortical circuits using home cages
Source: eLife. 2020 May 15;9:e55964. doi: 10.7554/eLife.55964 (PMC7332290; doi:10.7554/eLife.55964)
Supplement: Transparent reporting form [file elife-55964-transrepform.pdf]

Transparent reporting form

Automated task training and longitudinal monitoring of mouse mesoscale cortical circuits using homecages.

Sample-size estimation:

Our training environment consists of two major training procedures that needed to be developed, combined and verified within a low-cost Raspberry Pi based fully automated system:

- Self-initiated head-fixing
- and a reliable task-based training that yields brain imaging data comparable in quality and volume to manual head-fixation

Our sample size was chosen according to develop these procedures and confirm their reliability.

- 52 mice (44 males and 8 females)
- in 6 groups (1 female group and 5 male)
- of 8-10 mice each according to the guidelines of the Canadian Council for Animal Care

Since head-fixation is by default an aversive procedure (and with regard to our previous work - *Murphy et al., 2016)* we expected less than half of the mice to actually participate in the training. In order of having the ability to refine hardware and software **we tested 5 cages sequentially in an animal facility and a 6th separately in the lab**. After 6 cages we are certain that around ~40% of the mice within a group will reliably self-initiate head-fixation over long periods with our new methods.

Additionally, to verify the power of the system to establish a task-based training under self-initiated head-fixation and brain imaging, animals were recorded over 30 -90 days. Within this time we usually expect another mouse of each group to stop performing or experience issues with their windows. After 6 cages we are also certain that the majority of mice, once head-fixing, were participating in the task producing the intended amount of brain image data. 25/52 mice performed self-initiated head-fixation over multiple days

Replicates:

- The experiment was repeated 6 times by establishing a cage with 8 or 10 mice
- Each cage had at least 3 mice initially engaging head-fixation over multiple days
- Non-participating animals were removed and used for other experiments, further exclusion, though rare, was just done with regard to the health of the animal
- Experiments are 24/7 hour based and animals freely engage the task, which produces different amounts head-fixes for each day and mouse
- Experiments were usually lasting 50 – 120 days, while the first (approximately) 20 days are without full head-fixation, producing just behavioral data, which was usually not considered in figures, since head-fix data is priority in this method paper
- Once head-fixed, the remaining mice on average engaged the task 27 times per day producing 15 minutes of brain data each (male mouse stats). All together the active male mice initiated 33087 head-fixes (362.4 hours) each having multiple repeats of Go (or No-Go) task experiments within a head-fix session (male mouse data).

- Behavioral analysis isn't excluding data unless due to categorical selections (stated in figures) or in very rare cases of technical issues like a broken licking sensor or power outages
- Brain imaging shown for Go or No Go trials is done for specific dates and mice with regard to relevance or good quality. Example figures show 3 different mice which experienced Go and No Go trials
- Brain images exclude data of trials that are incomplete, or suffer from high standard deviation in brain fluorescence (attributed to non-head-fix trials). Threshold is set by user and then exclusion is automatically done by the software.

Statistical reporting:

Behavioral Analysis

- Behavioral analysis was done using a relational database (MySQL) for specified selection of data
- An interpretation script fills the database automatically from the raw data in various tables including specified information about each entry, trial, lick, reward and video. Raw data is generated by the running head-fix program and printed to text files. Raw data always include a tuple of: Mouse, Event, Timestamp
- For further analysis the database is used to select data for statistical tests and figures due to criteria like dates, mice, outcomes, head-fixations…
- Database can be publically accessed via repositories cited in the data. Additionally we provide a SQL dump
- Behavioral analysis and acquisition code can be found at GitHub (DavidBierbrauer and ubcbraincircuits/AutoHeadFix) and includes the queries and python code. Code can be run live by accessing our database to reproduce or explore figures, numbers and statistical tests. This also allows immediate analysis of future cage data
- Counted numbers were provided by the database using the corresponding queries and therefore were not estimated or rounded. Queries can also be found on the GitHub (female mouse data https://doi.org/10.5683/SP2/9RFXRP) and https://doi.org/10.5281/zenodo.3243572 male mouse.
- Statistical tests are done with common python libraries like statsmodels or scipy and used tests are mentioned in the paper whenever they appear. Please visit our methods section
- 1D kernel density estimation was used for clustering. This method is used by the community for example for spike density estimation and is therefore an established method

Image Analysis

- Videos were recorded with up to 4 cameras (1 brain, 3 behavior: face, eye, bottom) for each head-fix session (some sessions may only have 2 behavior cameras)
- Video analysis was done in MATLAB and provides different tools like behavior and brain image correlation, selectable seed pixel correlation, and time resolved averaging of brain activity profiles

- Brain images are averaged over multiple days, head-fixes and trials and are done for each mouse and classified by each outcome of trials for comparison and in case of successful go trials also with regard to degree of hind limb movement, 3 example mice are shown where image data was collected, all mouse image data is potentially available.
- Data for figure 8 are for cage 4 only, since these mice experienced Go and No Go trials and the longest lick delay times, we include .mat files with the processed images on Zenodo as a DOI https://doi.org/10.5281/zenodo.3243572, figures 9 and 10 include multiple cages as indicated all data files and code for figures 9 and 10 are found in https://doi.org/10.5683/SP2/ZTOPUM
- Raw video files together exceed 20 TB. We provide the processed data (.mat files) used in the MATLAB analysis with this submission. Raw video data can be provided on request
- Time binning was done pixel wise as indicated and uses maximum dF/F values for task activation images. MATLAB code can potentially be easily adjusted for average or minimum binning. Spatial binning was done to reduce 256x256 pixel raw videos of brain activity to 64x64 pixel processed images

Source Data

All text file behavioral data is included online as well as image data for figures 8 and supplemental figure 2 are found on https://doi.org/10.5281/zenodo.3243572, all data files and code for figures 9 and 10 are found in https://doi.org/10.5683/SP2/ZTOPUM and female mouse behavioral data https://doi.org/10.5683/SP2/9RFXRP. All Python data acquisition code can be found on https://github.com/jamieboyd/AutoHeadFix/ and https://github.com/ubcbraincircuits/AutoHeadFix.

Github repositories for code:

- jamieboyd (homecage software)
- https://github.com/ubcbraincircuits/AutoHeadFix (latest acquisition software)
- DavidBierbrauer (behavioral analysis)
